# Active travel and paratransit use in African cities: Mixed-method systematic review and meta-ethnography

**DOI:** 10.1016/j.jth.2022.101558

**Published:** 2023-01

**Authors:** Lee Randall, Anna Brugulat-Panés, James Woodcock, Lisa Jayne Ware, Caitlin Pley, Safura Abdool Karim, Lisa Micklesfield, Gudani Mukoma, Lambed Tatah, Philip Mbulalina Dambisya, Sostina Spiwe Matina, Ian Hambleton, Gabriel Okello, Felix Assah, Megha Anil, Haowen Kwan, Alice Charity Awinja, Georgina Pujol-Busquets Guillén, Louise Foley

**Affiliations:** a SAMRC/Wits Centre for Health Economics and Decision Science – PRICELESS-SA, School of Public Health, University of the Witwatersrand, Johannesburg, Gauteng, South Africa; b MRC Epidemiology Unit, University of Cambridge, Cambridge, United Kingdom; c SAMRC-Wits Developmental Pathways for Health Research Unit, Faculty of Health Sciences, University of the Witwatersrand, Johannesburg, Gauteng, South Africa; d DSI-NRF Centre of Excellence in Human Development, University of the Witwatersrand, Johannesburg, Gauteng, South Africa; e School of Clinical Medicine, University of Cambridge, Cambridge, United Kingdom; f Health Policy and Systems Division, School of Public Health and Family Medicine, University of Cape Town, Cape Town, South Africa; g George Alleyne Chronic Disease Research Centre, Caribbean Institute of Health Research, The University of the West Indies, Bridgetown, Barbados; h Cambridge Institute for Sustainability Leadership, University of Cambridge, Cambridge, United Kingdom; i Health of Populations in Transition (HoPiT) Research Group, Faculty of Medicine and Biomedical Sciences, The University of Yaoundé I, Yaoundé, Cameroon; j Adaptive Management Research Consultancy, Kisumu, Kenya; k Division of Exercise Science and Sports Medicine, Department of Human Biology, Faculty of Health Sciences, University of Cape Town, Cape Town, South Africa; l Faculty of Health Sciences, Universitat Oberta de Catalunya (Open University of Catalonia, UOC), Barcelona, Spain

**Keywords:** Paratransit, Active travel, African cities, Public health, Systematic review, Meta-ethnography

## Abstract

Active travel, as a key form of physical activity, can help offset noncommunicable diseases as rapidly urbanising countries undergo epidemiological transition. In Africa a human mobility transition is underway as cities sprawl and motorization rises and preserving active travel modes (walking, cycling and public transport) is important for public health. Across the continent, public transport is dominated by paratransit, privately owned informal modes serving the general public. We reviewed the literature on active travel and paratransit in African cities, published from January 2008 to January 2019. We included 19 quantitative, 14 mixed-method and 8 qualitative studies (n = 41), narratively synthesizing the quantitative data and meta-ethnographically analysing the qualitative data. Integrated findings showed that walking was high, cycling was low and paratransit was a critical mobility option for poor peripheral residents facing long livelihood-generation journeys. As an indigenous solution to dysfunctional mobility systems shaped by colonial and apartheid legacies it was an effective connector, penetrating areas unserved by formal public transport and helping break cycles of poverty. From a public health perspective, it preserved active travel by reducing mode-shifting to private vehicles. Yet many city authorities viewed it as rogue, out of keeping with the ‘ideal modern city’, adopting official anti-paratransit stances without necessarily considering the contribution of active travel to public health. The studies varied in quality and showed uneven geographic representation, with data from Central and Northern Africa especially sparse; notably, there was a high prevalence of non-local authors and out-of-country funding. Nevertheless, drawing together a rich cross-disciplinary set of studies spanning over a decade, the review expands the literature at the intersection of transport and health with its novel focus on paratransit as a key active travel mode in African cities. Further innovative research could improve paratransit's legibility for policymakers and practitioners, fostering its inclusion in integrated transport plans.

## Highlights

• Noncommunicable diseases are rising in Africa as populations urbanise and motorise.
• To offset this, active travel (walking, cycling, public transport) is desirable.
• Paratransit (informal public transport) is a key mode for urban African residents.
• Despite limited formal public transport many city authorities are anti-paratransit.
• Further paratransit research can foster more effective integrated mobility systems.

## 1 Introduction

From 1950 to 2018 Africa's urban population rose more than sixteen-fold, increasing from 33 million to 548 million; major African cities will double by 2050 due to population growth (high birth rates), international migration and movement from rural to urban areas (United Nations Department of Economic and Social Affairs Population Division], 2019). Developments include mega-cities (like the Gauteng City Region in South Africa) and meta-cities spanning national borders, like the four-country Ibadan-Lagos-Porto-Novo-Lomé-Accra corridor in Western Africa. Such ‘turbo-urbanization’ contributes to mobility challenges, as travel demands surpass the capacity of existing transport systems (Bruun and Behrens, 2016).

Formal public transport systems in many African cities were shaped by colonialism (and apartheid in South Africa), historically comprising limited bus and rail services catering to privileged groups rather than the population at large. Rapid urbanization has been characterized mainly by city sprawl with unplanned high-density peripheral settlements, with resulting trip distances necessitating at least some motorized transport as residents pursue livelihoods and fulfil household needs. In the post-colonial, post-apartheid era formal public transport has declined markedly (Klopp and Mitulla, 2016) and a human mobility transition has begun, strongly characterised by privatisation (private ownership) and commodification of transport (de Sá et al., 2019), with profit-driven selling of mobility services to city residents. Although car and motorbike ownership remain lower than in other world regions they are rising rapidly due to middle class aspirations and vehicle manufacturers’ targeting of Africa as a lucrative untapped market (Mokonyama and Venter, 2007). Some large African cities have introduced new (often foreign-funded) modes like Bus Rapid Transit (BRT) and Light Rail, based on their success in Latin America and Asia, but mobility systems broadly are dysfunctional, with rising traffic congestion and pollution. Consequences include transport inequity (Foley et al., 2022) and threats to sustainability and quality of life (United Nations Environment Programme, 2020).

Alongside the mobility transition Africa is undergoing an epidemiological transition characterised by an increase in noncommunicable diseases in addition to the established communication diseases. In tandem with this transition is a rise in physical inactivity (Guwatudde et al., 2016; Lear et al., 2017; Milton et al., 2014). Preserving moderate to vigorous physical activity improves individual and population health (Katzmarzyk et al., 2021) and transport-related physical activity can contribute significantly to the 150 min per week recommended by the World Health Organisation (Bartels et al., 2016). Promoting non-car trips can create significant health benefits (Langlois et al., 2016), making it important to limit mode-shifting to private vehicles and away from active travel (walking, cycling and use of public transport). However, a physical activity paradox is evident, especially in relation to occupational rather than leisure activity (Holtermann et al., 2021) – thus, health-harms may arise from onerously long journeys on foot, especially over hostile terrain or whilst load-carrying (Laverty et al., 2015; Nkurunziza et al., 2010; Porter et al., 2013). A recent modelling study from Accra (Ghana) found no net health benefits from substituting short car journeys with walking or cycling, as disease prevention was outweighed by increased road traffic deaths; by contrast, net health benefits resulted from substituting *private* with *public transport* (Garcia et al., 2021). Maximising health benefits and minimising health harms associated with active travel in African cities requires an optimal mix of travel modes, including non-motorized for shorter journeys and motorized for longer journeys.

With respect to public transport, in African cities a significant share consists of paratransit, or informal, unscheduled, privately owned transport services, generally offered by small-scale operators on a for-profit basis. Paratransit's share of road-based public passenger transport ranges from 65% in Yaoundé (Central Africa) and 72% in Johannesburg (Southern Africa) to 82% in Algiers (Northern Africa), 86% in Accra (Western Africa) and 98% in Dar es Salaam (Eastern Africa) (Behrens et al., 2016). Paratransit types include motorized four-, three- and two-wheelers as well as non-motorized rickshaws, bicycle taxis and animal-drawn carts. Together they outweigh the mode shares of formal public transport (buses, trains and BRT). United Nations Sustainable Development Goals SDG3 (“Good health and well-being”) and SDG11 (“Make cities inclusive, safe, resilient and sustainable”) rest in part on active travel and public transport, with Target 11.2 particularly relevant: “By 2030, provide access to safe, affordable, accessible and sustainable transport systems for all, … notably by expanding public transport, with special attention to the needs of those in vulnerable situations, women, children, persons with disabilities and older persons” (United Nations General Assembly, 2015). From a sustainable development and epidemiological perspective paratransit is of significant interest but has not previously been researched in relation to active travel and public health. This knowledge gap may relate to city authorities not formally recognizing it, failing to regulate it and having anti-paratransit policies like the central city Boda-Boda Free Zone in Kampala, Uganda (KCCA, 2020).

Our mixed-method systematic review and meta-ethnography, couched within the public health domain, used a physical activity/active travel lens to examine the literature from across transport, health and social sciences disciplines to explore paratransit use in African cities. The research questions we sought to answer were:

1. What factors are associated with the use of paratransit in rapidly growing African cities, and how have these factors contributed to the proliferation of paratransit modes?
2. How does paratransit use intersect with other active travel modes (walking, cycling and use of formal public transport) as well as non-active travel modes (use of private cars and motorbikes)?
3. How do residents choose between and experience different travel modes?
4. What paratransit interventions could help maximise active travel and minimise travel harms in African cities?

## 2 Methods

### 2.1 Search strategy and selection criteria

We drew our review from a parent review of a larger set of literature published from 1 January 2008 to 31 January 2019 and examining factors associated with regular or routine travel behaviour in Africa and the Caribbean (PROSPERO registration number CR42019124802).^1^ We followed PRISMA (Preferred Reporting Items for Systematic Reviews and Meta-Analyses) (Moher et al., 2009) and eMERGe (France et al., 2019) guidelines. For the parent review we searched MEDLINE, Transport Research International Documentation (TRID), SCOPUS, Web of Science, LILACS, SciELO, Global Health, Africa Index Medicus, CINAHL and MediCarib to cover the most relevant disciplines (transport, health and social sciences) and geographical focus, piloting the search in December 2018 and completing it in February 2019 in consultation with a medical librarian - full search strategy available elsewhere (Foley et al., 2022). We used Covidence software^2^ to import citations and remove duplicates, then paired junior team members performed title-and-abstract screening and full-text screening with a senior team member serving as arbiter. We added citations mined from literature reviews, forward searching (citation screening using Scopus and Web of Science) and backward searching (screening of reference lists of eligible studies), after screening by a senior team member. We also identified 27 topic experts (authors of multiple eligible studies) and in November 2020 invited them to provide additional citations; eight did so and a senior team member screened them and imported eligible ones into Covidence.

This review included studies in urban African settings (eligible countries listed in Supplementary File 1) with a focus on active travel and paratransit use. We defined active travel as walking, cycling or using public transport – formal or informal – to get from one place to another for purposes other than purely recreational. We deemed public transport use as active travel due to some walking or cycling typically being required (Costa et al., 2015) and defined paratransit as unscheduled, flexible public passenger transportation, falling between autonomous private transport and scheduled, fixed-route public transport (Behrens et al., 2016). Due to varying nomenclature we selected studies overtly containing the term paratransit and studies containing terms like *informal public transport* and *unscheduled public transport*, as well as those containing words denoting specific paratransit modes. During our first-pass scrutiny of potentially eligible studies we assembled a non-exhaustive list of countries’ paratransit naming conventions, commonly reflecting historical or mode characteristics – for instance, *boda-bodas* crossed national borders, *bendskins* were known for speed and *dala-dalas, trotros* and *okadas* were named after their early fares. Our search strategy included colloquialisms for minibus taxis (eg. *matatu* in Kenya (Mutiso and Behrens, 2011; Salon and Gulyani, 2010), *kombi* in South Africa (Lucas, 2011), *trotro* in Ghana (Saddier et al., 2016), *daladala* in Tanzania (Andreasen and Møller-Jensen, 2017) and *chapas* in Mozambique (Tembe et al., 2018), motorcycle taxis (*boda-boda* in Eastern Africa (Andreasen and Møller-Jensen, 2017; Porter et al., 2013) and *moto-taxi*, *okada* or *bendskin* in Western Africa (Esson et al., 2016; Kumar, 2011)), three-wheelers (*tuk-tuk* in South Africa (Mutiso and Behrens, 2011), *kekenapep* in Nigeria (Nwaogbe et al., 2012),*toc-toc* in Egypt (Elfiky, 2010), *bajaj* in Tanzania (Andreasen and Møller-Jensen, 2017)) and bicycle taxis (*bicycle boda* or *boda-boda* in various countries (Janusz et al., 2019; Kola et al., 2012; Kumar, 2011; Mutiso and Behrens, 2011; Turner and Adzigbey, 2012)). Studies had to contain empirical data (primary or secondary) with an analysis, and all study designs (quantitative, qualitative and mixed-method) were eligible. We excluded literature reviews, narrative overviews, commentaries, opinion pieces and citations which provided insufficient information to allow for data extraction. Included studies focused on urban African populations, with no age or sex restrictions but with exclusion of studies focused on specific or unique population segments in which travel was likely to be atypical. This included people with health conditions or impaired mobility (other than age-related impairment), professional travellers (eg: bus or *matatu* drivers), tourists, refugees, asylum seekers, migrants and victims of trafficking. Studies were also excluded studies if their focus was non-human travel (eg: food, freight). With regards to exposures, we included both correlates (where causality was uncertain) and purported causal influences on travel behaviour. Any comparators were eligible, if used. The outcomes of interest were aspects of regular or routine travel behaviour including at least some paratransit use; these were time spent in all travel or particular travel modes; number of trips; choice or use of particular travel modes or combinations of modes; and mode share. We excluded studies focused on single travel purposes (school-related travel, travel to administer or receive healthcare) and on hypothetical (rather than actual) use of transport modes. Studies were also excluded if they lacked a primary focus on travel *per se* – for instance, those focused on road traffic injuries as the main outcome – or were set in contexts in which travel was likely to be atypical, like war, political crises and natural disasters. All languages were eligible.

### 2.2 Data extraction

We customised, piloted and refined a Covidence data extraction template (Supplementary File 2) which included fields for authors and their affiliations, funding sources, geographic setting, dates of data collection, publication year, research design, methods, participants, exposures, outcomes and results. Data were initially extracted by trained junior team members, with senior members double-extracting selected fields for a random 20% of the studies. Unacceptable inconsistency (>50%) appeared to relate to the complex review topic and study heterogeneity, causing senior team members LR, LF and ABP to repeat data extraction for all studies (resolving difficulties via discussion and consensus). We made reflexive notes on Trello,^3^ an online collaboration and project management platform; these were available to all team members and enhanced our contextual sensitivity.

### 2.3 Study quality appraisal

We subjected all studies to quality appraisal using Critical Appraisal Skills Programme (CASP) checklists^4^ to assess risk of bias, appropriateness of research design and recruitment strategy, analytical rigour (including controlling for confounding variables where appropriate) and effectiveness of result-reporting. For quantitative studies and quantitative aspects of mixed-method studies we used an adapted CASP cohort study checklist and for qualitative studies and qualitative aspects of mixed-method studies we used the CASP qualitative checklist. For purposes of our meta-ethnography we also performed a bespoke ‘thickness’ rating using Ponterotto's five thickness components: interpretation in context, capturing of participants' thoughts and emotions, verisimilitude (making the setting ‘come alive’ for readers), ascribing of motivations and intentions to participants, and explaining the meaningfulness of situations and of the research findings (Ponterotto, 2006). We rated each component on a scale of 1–4 (1 = minimal thickness and 4 = maximal thickness) and summed the totals to give an overall thickness score ranging from 5 (minimal thickness on all components) to 20 (maximal thickness on all components). We piloted this rating scale on two randomly selected studies, one qualitative and one mixed-method, and found good inter-rater consistency between LR, LF and ABP. We did not exclude any studies for low thickness but our ratings helped us identify those which were particularly rich and well-contextualised; re-reading of these improved our insights in relation to other studies with similar foci or geographic settings.

### 2.4 Data syntheses and integrated analysis

We used a sequential explanatory method (Fetters et al., 2013), synthesizing first the quantitative data and then the qualitative data. We examined the coherence between the syntheses as a process of ‘holistic sense making’ (Ogilvie et al., 2020).

For our quantitative synthesis we entered the study results into an Excel spreadsheet and summarised them narratively. Mathematical aggregation was not possible due to heterogeneity in the study outcomes and units of measurement – some measured human parameters (eg: purpose of travel, reasons for mode choice, satisfaction levels) while others focused on geographical-technical parameters (eg: routes, GPS tracks, inbound and outbound traffic units) as proxies for human travel behaviour. We captured results in terms of direction of effects rather than magnitude or causality, both of which were frequently not provided. To evaluate the certainty of the evidence we drew on the Bradford Hill domains of consistency (similar findings across multiple settings and studies), coherence (similar findings across different disciplines and methods) and analogy (similarities between related findings) (Hill, 1965). We did this through team discussion and by consensus we categorized the evidence as *compelling* where a result appeared in more than 50% of the studies, *clear* where it appeared in 25–49%, *fair* where it appeared in 10–24% and *sparse* where it appeared in fewer than 10%.

Our synthesis of the qualitative data used a meta-ethnographic approach (Ponterotto, 2006). We entered second-order constructs (ie. study authors’ interpretations of their primary data) (Toye et al., 2014) on a customized Excel spreadsheet after piloting this using the same two studies on which the thickness rating scale was piloted. This was an immersive process during which we re-read the studies and also referred back to our reflexive notes on Trello. During a virtual meeting LR, LF and ABP compared (translated) the studies against each other, repeatedly scanning the second-order constructs to identify reciprocal and refutational relationships. Through this highly iterative process we grouped them into what appeared to be logical clusters and gave each cluster a working title, representing a tentative third-order construct or theme, namely our reviewer interpretations of the aggregation of findings across studies (Toye et al., 2014).

During a virtual meeting with the full research team the quantitative synthesis was presented first, followed by the preliminary meta-ethnographic findings. We used Jamboard^5^ to visually depict the early meta-ethnographic clusters and the team sense-checked, critiqued and refined these. Through discussion and consensus some clusters were merged and others split, with the final clusters retitled as needed – for instance, the working title “Transport as a basic need and right” was replaced with “Transport as an enabler”. In this way the preliminary meta-ethnography was refined with input from team members with different disciplines (chiefly health, social sciences and transport) and geographic backgrounds (including multiple African countries). Some with subject-matter expertise and/or first-hand knowledge of the study settings noted unexpected gaps in the data, allowing LR, LF and ABP to revisit the studies after the meeting and check for missed second-order constructs (there were none). The team meeting culminated in a discussion regarding the coherence between the quantitative and qualitative syntheses, in the spirit of Trochim's complex pattern-matching process (which conventionally determines whether an observed pattern matches a hypothesized pattern) (Trochim, 1989). Where strong coherence was identified we found this to be an indication that the qualitative data had explanatory strength in relation to the quantitative data.

Our final analysis was enriched by stakeholder engagement within the Global Diet and Activity Research (GDAR) network^6^ (Oni et al., 2020) and with key informants in prominent international organisations, namely the World Health Organisation, UN Human Settlements Program (UN-Habitat) and the Institute for Transportation and Policy Development (ITDP). These key informants reflected on our emergent findings and their implications for policy, practice and future research during separate online meetings in February/March 2021.

## 3 Results

### 3.1 Included studies

**Fig. 1:**
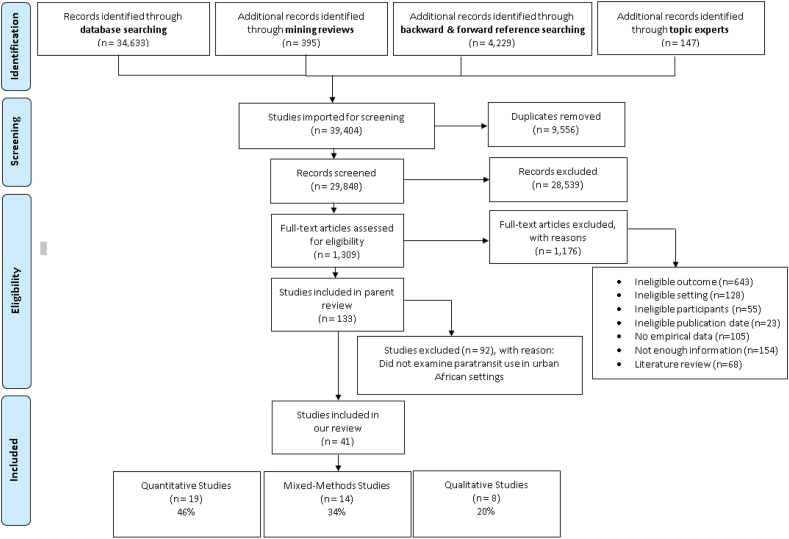
PRISMA flow diagram for studies retrieved through the searching and selection process.

From 39,404 citations, 133 studies met eligibility criteria for the parent review and a subset of 41 studies met eligibility criteria for our review (see Supplementary file 3). Of these, 19 (46%) were quantitative, 14 (34%) were mixed-method and 8 (20%) were qualitative – see PRISMA flow diagram (Fig. 1). All the quantitative studies were cross-sectional, involving surveys, interviews and intercept studies. Some contained primary or re-processed secondary data from human subjects, with widely varying sample sizes (n = 10 to n = 20,000+) while others used non-human measurements like trips, GPS tracks and in- and out-bound traffic units. The qualitative studies were predominantly ethnographic or phenomenological. Most studies did not overtly name an underpinning theory but those which did drew on the mobilities (or “new mobilities”) paradigm with its focus on power relations (Porter et al., 2017), a social exclusion perspective (Alando and Scheiner, 2016; Lucas, 2011), Hägerstrand's space-time prism (Janusz et al., 2019; Vermeiren et al., 2015), political economy (Kumar, 2011) or development studies perspectives (Poku-Boansi and Cobbinah, 2018), social quality theory (Alando and Scheiner, 2016), a person-centred framework linking transport and different dimensions of wellbeing (material, relational and subjective) (Oviedo et al., 2017), and Kaufmann's typology of four forms of mobility (daily mobility, residential mobility, migration and travel) with his notion of ‘motility’ reflecting rational, socio-geographical and sociopsychological factors (Irlam and Zuidgeest, 2018; Yankson et al., 2017).

### 3.2 Geographic representation

**Fig. 2:**
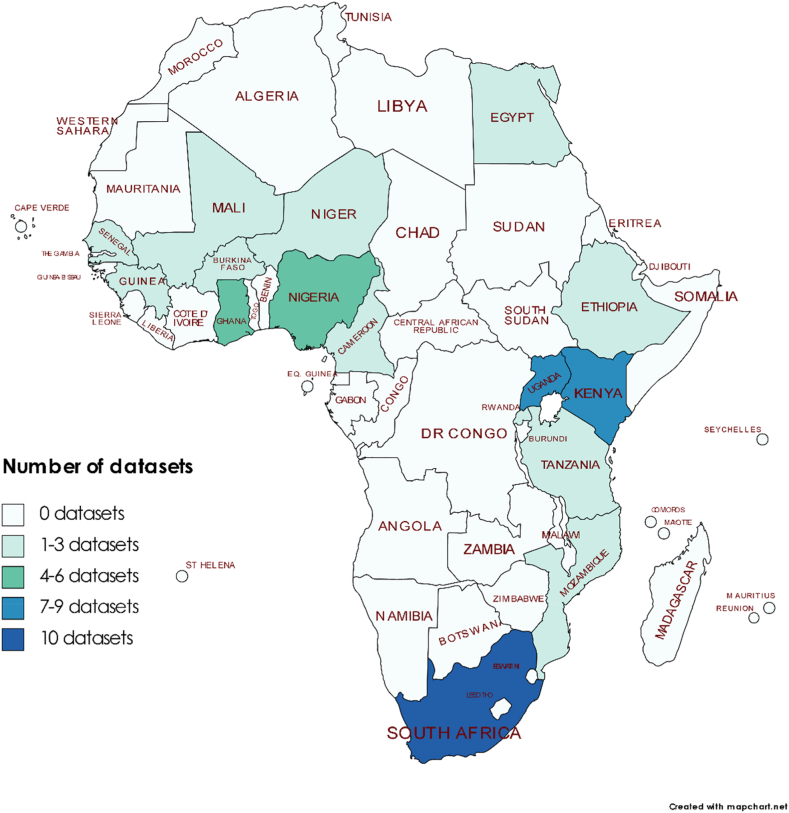
Geographical distribution of datasets included in paratransit analysis Created using https://mapchart.net/. Licensed under a Creative Commons Attribution-ShareAlike 4.0 International License.

Some studies were multi-country and others multi-region, yielding 64 datasets in all. These were drawn from 16 (30%) of the 54 countries in Africa, with some countries appearing multiple times but most not showing up in the data at all. Regional and intra-regional representation was highly uneven, with only two datasets (3%) respectively from Northern Africa and Central Africa; both the former were from Egypt and both the latter were from Cameroon. By contrast, 17 (27%) of the datasets related to countries in Western Africa (Ghana 4, Nigeria 6, Senegal 2, Burkina Faso 1, Guinea 1, Mali 1 and Niger 2), while 21 (33%) related to countries in Eastern Africa (Ethiopia 1, Rwanda 1, Uganda 7, Tanzania 2, Kenya 8 and Mozambique 2). Southern Africa appeared relatively well represented, with 10 (16%) of the datasets; however, all were from a single country (South Africa). The geographical distribution of the datasets is shown on a choropleth map (Fig. 2). In total, 32 cities or city regions appeared in the data, with some strongly represented. For instance, Kampala (Uganda) and the Gauteng City Region in South Africa (including Johannesburg and Pretoria/Tshwane) each featured seven times (making up 11%, respectively, of the datasets) while Nairobi (Kenya) featured five times (making up 8% of the datasets). In all, 44 (69%) of the datasets related to capital or former capital cities.

### 3.3 Types of paratransit reflected in the data

We sorted the paratransit types which showed up in the data, including both those which were a primary focus of the researchers and those which were mentioned (sometimes described in depth) as part of the overall paratransit system in the research setting. We grouped them according to their common features, revealing eight distinct categories (Supplementary Table 1). Motorized types were most common, including four-wheelers, two-wheelers and three-wheelers. Nonmotorized types, less frequently reflected, included bicycle taxis and three-wheelers as well as horse-drawn carts. Twenty two studies (54%) mentioned only one type of paratransit without indicating whether this was the only type present in that setting or just the one which attracted the researchers’ gaze. By contrast, 9 (22%) of the studies mentioned 3–4 paratransit types. It was not possible to perform a thorough regional breakdown given that some studies were multi-regional and did not specify paratransit types per region. Overall, minibus/midibus taxis made up 90% of the paratransit types reported in Southern African studies, 47% in Western African studies, 45% in Eastern African studies and 25% in Northern African studies. Motorbike taxis made up 27% of the paratransit types reported in Western African and 29% in Eastern African studies but were absent in mono-regional studies from other regions. Three-wheelers (motorized or unmotorized) dominated Northern African studies, making up 75% of the paratransit types mentioned; they also appeared in Western and Eastern African studies (20% and 10% respectively). Bicycle taxis appeared only in Eastern African studies, making up 13% of paratransit types mentioned, shared sedan (car) taxis were specific to Eastern Africa (3%) and adapted trucks were specific to Western Africa (7%).

### 3.4 Author affiliations and funding sources

**Fig. 3:**
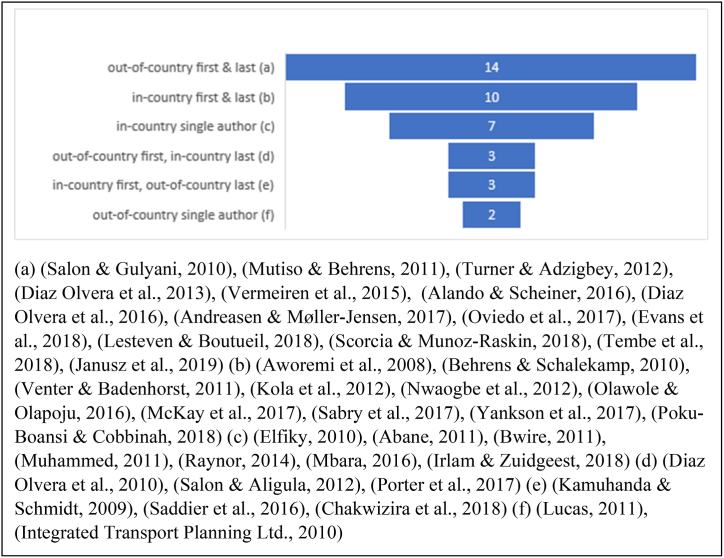
Single, first and last author affiliations - excluding two studies (Kumar, 2011; Weinstock et al., 2015) which were carried out, respectively, by regional and international organisations.

We noted a phenomenon of out-of-country author affiliation with 16 (39%) of the studies having single or senior (first or last) authors affiliated to institutions in countries other than where the research occurred (Fig. 3). Most out-of-country authors were affiliated to institutions in high-income countries in the European region (UK 6, France 4, Belgium 2, Germany 2, Denmark 1); others had affiliations in the USA (3) and Japan (1). Over half of the studies (22 or 54%) reported funding sources and some had multiple sources. Four (10%) were funded by the Volvo Research & Educational Foundations (Behrens and Schalekamp, 2010; Bwire, 2011; Mutiso and Behrens, 2011; Salon and Aligula, 2012), one (2%) was funded the African Development Bank and World Bank (Kumar, 2011) and three (7%) were funded by international organisations (UN-Habitat, ITDP, National Geographic and International Labor Organization) (Evans et al., 2018; Kamuhanda and Schmidt, 2009; Salon and Gulyani, 2010). Ten (24%) reported other out-of-country funding, including from donor agencies in France, Belgium, Denmark, Germany, Ireland, Norway, Sweden, the European Union, the United Kingdom, Canada and the United States of America (Alando and Scheiner, 2016; Andreasen and Møller-Jensen, 2017; Lesteven and Boutueil, 2018; Oviedo et al., 2017; Porter et al., 2017; Saddier et al., 2016; Salon and Aligula, 2012; Salon and Gulyani, 2010; Vermeiren et al., 2015; Yankson et al., 2017). Only five (12%) reported funding from the government of the country in which the research took place (Alando and Scheiner, 2016; Diaz Olvera et al., 2016; Lucas, 2011; McKay et al., 2017; Venter and Badenhorst, 2011).

### 3.5 Quality appraisal

For the quantitative data (drawn from 33 quantitative and mixed-method studies) we found possible bias in 18 (55%) of the studies, rating them as either deficient or highly non-informative when it came to considering and controlling for confounding factors. We rated recruitment strategies as deficient or highly inadequately described in 9 (27%) of the studies. The CASP checklist criteria suggested a lack of objective and accurate measurement of exposures and outcomes in 7 (35%) of the studies; however, while CASP penalises use of self-reported data we noted that such data is commonly used (and accepted) in physical activity and travel behaviour research.

For the qualitative data (drawn from 22 qualitative and mixed-method studies) we rated 19 (86%) of the studies as deficient or highly non-informative in relation to ethical clearance, use of consent forms and/or measures taken to ensure confidentiality. We found that 7 (32%) described participant recruitment strategies poorly or not at all. We rated 15 (68%) as deficient or highly non-informative with regards to the relationship between researchers and participants; there was a general lack of reflexivity, with uneven power relations and language and cultural differences between researchers and participants largely unaddressed. In relation to rigour 15 (68%) of the studies did not clearly describe how the data were analysed. Overall, we thus had significant concerns about the quality of the studies but in many instances this was due to missing information rather than positive indications of poor or unethical methods or inadequate analysis. We did not exclude any studies on grounds of quality and the degree to which poor study quality may have affected our qualitative findings is unknown. Our bespoke thickness rating method showed that many studies lacked thickness, with total scores ranging from 6 to 19 (possible score range = 5–20); mean scores were 11.8 for qualitative studies and 10.8 for mixed-method studies. Thickness component “capturing thoughts and emotions” was rated lowest on a scale of 1–4, with study means of 1.9 (qualitative) and 1.5 (mixed-method). Highest-rated thickness components were “interpretation in context” and “explaining the meaningfulness of situations and the results”, in both cases yielding means of 2.9 (qualitative) and 2.7 (mixed-method). Intermediate scores were assigned to “verisimilitude” (2.0 and 1.8) and “ascribing motivations and intentions” (2.3 and 2.1).

### 3.6 Quantitative findings

Our findings with respect to the quantitative data appear in Table 1. [14]

**Table 1:** Narrative synthesis of quantitative findings.

Domain	Detailed findings and studies in which they appeared	Certainty of evidence	Number of studies *n* (%)
Intersections between paratransit and other travel modes (active and non-active):			
Intersection between paratransit and walking	Paratransit and walking were substitutional: walking was the most common and often the only mode available, especially for children and females, but residents with the means to pay fares used paratransit, especially for longer journeys. When funds allowed they favoured paratransit over walking even for shorter trips, making it an aspirational mode with walking as a fall-back (Andreasen and Møller-Jensen, 2017; Aworemi et al., 2008; Diaz Olvera et al., 2010, 2013; Elfiky, 2010; Integrated Transport Planning Ltd, 2010; Irlam and Zuidgeest, 2018; Kola et al., 2012; Kumar, 2011; Mutiso and Behrens, 2011; Olawole and Olapoju, 2016; Salon and Aligula, 2012; Tembe et al., 2018; Venter and Badenhorst, 2011).	*clear evidence*	14 (42%)
Intersection between paratransit and formal public transport	Paratransit improved residents' access to formal public transport, serving as a gap-filler and offering last-mile, first-mile and feeder services in relation to buses, trains and Bus Rapid Transit. These formal services had lower mode shares than paratransit, with their shares boosted by paratransit connecting residents to them (Andreasen and Møller-Jensen, 2017; Diaz Olvera et al., 2010, 2016; Kumar, 2011; Mbara, 2016; Mutiso and Behrens, 2011).	*fair evidence*	6 (18%)
Intersection between paratransit and cycling	There was no real interface between paratransit use and cycling, with the latter having a very low mode share and being confined largely to poorer residents, males and children; it was often stigmatized as a marker of poverty, seen as unsafe and perceived as inaccessible due to costs and physical ability requirements (Elfiky, 2010; Integrated Transport Planning Ltd, 2010; Irlam and Zuidgeest, 2018; Sabry et al., 2017; Salon and Aligula, 2012; Venter and Badenhorst, 2011). In some settings cycling was seen as an alternative to using paratransit modes (Alando and Scheiner, 2016).	*fair evidence*	6 (18%)
Intersection between paratransit and non-active travel using private vehicles (cars and motorbikes)	Paratransit compensated for low ownership of private vehicles (cars and motorbikes), which was confined to wealthier residents and especially males. It also offered a back-up for private vehicle owners in times of vehicle breakdown or to save on running costs and sometimes served as a preferential option, obviating the need to find parking and allowing faster travel through traffic (Aworemi et al., 2008; Chakwizira et al., 2018; Diaz Olvera et al., 2013; Evans et al., 2018; Janusz et al., 2019; Kumar, 2011; Salon and Aligula, 2012; Venter and Badenhorst, 2011).	*fair evidence*	8 (24%)
Basis of travel mode choice:			
Socioeconomic status	Cost was a key driver of what modes were used, with income-poverty effectively removing choice and resulting in captive walking for many people (especially children, females and poorer residents) (Abane, 2011; Andreasen and Møller-Jensen, 2017; Chakwizira et al., 2018; Diaz Olvera et al., 2010, 2013; Integrated Transport Planning Ltd, 2010; Irlam and Zuidgeest, 2018; Janusz et al., 2019; Kumar, 2011; Muhammed, 2011; Olawole and Olapoju, 2016; Oviedo et al., 2017; Porter et al., 2017; Salon and Aligula, 2012; Salon and Gulyani, 2010; Venter and Badenhorst, 2011; Vermeiren et al., 2015).	*compelling evidence*	17 (52%)
Gender	Gender inequity was apparent, with females and males having different travel choices and experiences. Females were more reliant on public transport while males had greater access to private vehicles. Females were less likely than males to afford paratransit and also experienced it as less satisfactory, compared to males (Behrens and Schalekamp, 2010; Kumar, 2011; Mbara, 2016; Olawole and Olapoju, 2016; Oviedo et al., 2017; Porter et al., 2017; Sabry et al., 2017; Salon and Gulyani, 2010).	*fair evidence*	8 (24%)
Travel needs	Mode choices were shaped by locations of origins and destinations as well as trip distances. Residents' travel needs related to their employment status, gender and age profiles, household make-up, availability of private vehicles and possession of driver's licences; they were also shaped by personal habits and preferences and were altered by unusual circumstances (eg: bad weather, running late, night travel) (Abane, 2011; Andreasen and Møller-Jensen, 2017, Nwaogbe et al., 2012; Behrens and Schalekamp, 2010; Diaz Olvera et al., 2013; Evans et al., 2018; Integrated Transport Planning Ltd, 2010; Janusz et al., 2019; Kola et al., 2012; Oviedo et al., 2017; Sabry et al., 2017; Salon and Gulyani, 2010; Scorcia and Munoz-Raskin, 2018; Tembe et al., 2018; Venter and Badenhorst, 2011; Vermeiren et al., 2015).	*clear evidence*	16 (49%)
Access	Physical availability of modes in travellers' locations strongly dictated travel behaviour, with peripheral residents having fewer choices (access-poverty) and experiencing energy-poverty and time-poverty due to long, slow journeys to city centres. Lack of access to motorized modes resulted in captive walking, confinement to a small radius from home, or even complete immobility. (Abane, 2011; Andreasen and Møller-Jensen, 2017; Chakwizira et al., 2018; Diaz Olvera et al., 2013; Olawole and Olapoju, 2016; Salon and Aligula, 2012; Tembe et al., 2018; Venter and Badenhorst, 2011).	*fair evidence*	8 (24%)
Safety	Many residents reported concerns regarding road safety and personal security whilst travelling but these were not specific to paratransit; they did not influence mode choices in situations where there were few alternatives (Abane, 2011; Chakwizira et al., 2018; Kumar, 2011; Mbara, 2016; Venter and Badenhorst, 2011).	*fair evidence*	5 (15%)
Comfort and convenience	For those with the means to pay fares, mode choices were influenced by comfort and convenience, including the mode's reliability and ability to negotiate congestion speedily. This applied to choices between private vehicles, paratransit and formal public transport, as well as choices between different paratransit modes (eg: two-wheeled versus four-wheeled, one-passenger versus multi-passenger). (Andreasen and Møller-Jensen, 2017; Chakwizira et al., 2018; Evans et al., 2018; Integrated Transport Planning Ltd, 2010; Kola et al., 2012; Kumar, 2011; Mbara, 2016; Oviedo et al., 2017; Porter et al., 2017; Salon and Aligula, 2012; Venter and Badenhorst, 2011).	*clear evidence*	11 (33%)

Key to grading of evidence: *compelling =* result appeared in >50% of studies; *clear =* 25–49%; *fair =* 10–24%; *sparse=<*10%.

### 3.7 Qualitative findings

The qualitative data included contextual data regarding the African city backdrop against which paratransit modes operated, as well as broader travel behaviour data and narrower paratransit-specific data. As noted earlier, in keeping with accepted meta-ethnographic methods, we used an immersive and iterative process to aggregate second-order constructs from the studies and sort, group and re-group these (with input from the broader research team) in order to settle on a set of third order constructs – namely, our reviewer interpretations of the collective set of second-order constructs. This process yielded the following third-order constructs, which we chose to name Themes 1 to 6, giving each a unique name drawn from the literature. It was not possible or appropriate, for a meta-ethnography, to assign weights to the various second order constructs contributing to a particular theme, nor did we perform numerical analyses such as counting how many of the studies shaped each theme.

#### 3.7.1 Theme 1: The mobility gap in African cities

The selective focus and eventual collapse of colonial and apartheid transport systems, followed by neo-colonial road-building projects by governments and development banks, left many African cities with significant unmet travel needs (Alando and Scheiner, 2016; Lesteven and Boutueil, 2018; Lucas, 2011; Poku-Boansi and Cobbinah, 2018). There were spatial mismatches between where people lived and worked (Alando and Scheiner, 2016; Lucas, 2011), with long distances to key destinations (eg: workplaces, markets, educational and health facilities) making motorized transport necessary; high journey times and slow travel were common (Alando and Scheiner, 2016; Diaz Olvera et al., 2013; Lucas, 2011; Turner and Adzigbey, 2012). Unplanned and informal residential areas developed on city peripheries due to population growth and urbanisation(Andreasen and Møller-Jensen, 2017; Kola et al., 2012; Kumar, 2011; Lucas, 2011; Poku-Boansi and Cobbinah, 2018; Yankson et al., 2017) and residents of these dense “dormitory neighbourhoods” experienced severe travel challenges due to dysfunctional city mobility systems (Andreasen and Møller-Jensen, 2017; Oviedo et al., 2017; Poku-Boansi and Cobbinah, 2018; Turner and Adzigbey, 2012). Transport demand outstripped supply and street connectivity was poor due to fragmented land use- and transport-planning; many areas had no formal public transport at all, none within walking distance or none outside peak hours (Lucas, 2011; Turner and Adzigbey, 2012). The mobility gap was especially evident in cities serving “new economies” centred on oil, gas or rubber (which had changed travel needs) and intermediate and secondary cities were deprioritized compared with capital and mega-cities (Yankson et al., 2017). The mobility gap and forms of transport disadvantage were invisible to city officials (Alando and Scheiner, 2016; Yankson et al., 2017).

Residents' mobility needs and practices were diverse but all – even better-off residents in central areas – faced constrained mode choices (Abane, 2011; Andreasen and Møller-Jensen, 2017; Evans et al., 2018; Kola et al., 2012; Kumar, 2011; Vermeiren et al., 2015). Private vehicle ownership was low and motorability poor due to bad roads and rising congestion (Alando and Scheiner, 2016; Andreasen and Møller-Jensen, 2017; Diaz Olvera et al., 2013; Evans et al., 2018; Janusz et al., 2019; Kola et al., 2012; Poku-Boansi and Cobbinah, 2018; Yankson et al., 2017), sometimes aggravated by topography and climate (Alando and Scheiner, 2016; Evans et al., 2018; Irlam and Zuidgeest, 2018; Kumar, 2011). Sociocultural and gender norms limited travel options – for instance, cycling was largely confined to males (Alando and Scheiner, 2016; Irlam and Zuidgeest, 2018). Lack of mobility options affected residents’ ability to participate in livelihood activities and social networking, with the poorest groups systematically excluded from opportunities (Alando and Scheiner, 2016; Diaz Olvera et al., 2013; Janusz et al., 2019; Lucas, 2011; Oviedo et al., 2017; Yankson et al., 2017). Affordability was a key driver, with transport costs high relative to incomes and transport subsidies rarely available (Abane, 2011; Diaz Olvera et al., 2010, 2013; Evans et al., 2018; Janusz et al., 2019; Kumar, 2011; Lucas, 2011; Poku-Boansi and Cobbinah, 2018; Raynor, 2014; Vermeiren et al., 2015). Many urban poor were captive walkers (Diaz Olvera et al., 2013; Integrated Transport Planning Ltd, 2010; Irlam and Zuidgeest, 2018; Porter et al., 2017) but walkability was hampered by unpaved roads, potholes, lack of sidewalks, pedestrian crossings and streetlighting, and heavy vehicular traffic (Diaz Olvera et al., 2013; Integrated Transport Planning Ltd, 2010; Janusz et al., 2019; Turner and Adzigbey, 2012). Some residents were trapped on the periphery, with space-time prism studies revealing major limits to their feasible movement (Janusz et al., 2019). Travelling in general was perceived as unsafe but there were additional safety concerns related to mobility of women and girls (Abane, 2011; Irlam and Zuidgeest, 2018; Lucas, 2011; Mbara, 2016; Oviedo et al., 2017; Poku-Boansi and Cobbinah, 2018; Porter et al., 2017; Raynor, 2014; Turner and Adzigbey, 2012). Power relations in society shaped the mobility gap and perpetuated intergenerational cycles of poverty (Lucas, 2011; Yankson et al., 2017), with the gap manipulated for competitive advantage by transport operators and politicians (Abane, 2011). Factors which reduced it included mobile phones (substituting for physical travel) (Diaz Olvera et al., 2013; Porter et al., 2017) and new mobility practices like ride-hailing services (Uber), app-based trip planning (SafeBoda) and goods delivery options (Deliveroo) (Evans et al., 2018).

#### 3.7.2 Theme 2: Transport as an enabler

Transport was perceived as a basic need and its availability or non-availability as an equity and human rights issue (Alando and Scheiner, 2016; Diaz Olvera et al., 2013; Lucas, 2011; Oviedo et al., 2017). It enabled access to employment and livelihoods; education and training; healthy and affordable food; health care; and social, cultural and religious participation (Alando and Scheiner, 2016; Andreasen and Møller-Jensen, 2017; Diaz Olvera et al., 2013; Evans et al., 2018; Janusz et al., 2019; Kamuhanda and Schmidt, 2009; Lucas, 2011; Vermeiren et al., 2015; Yankson et al., 2017) as well as movement of messages and goods (Diaz Olvera et al., 2010; Evans et al., 2018; Kamuhanda and Schmidt, 2009). Motorized transport allowed residents to avoid overly long or unsafe walking trips (Diaz Olvera et al., 2013; Integrated Transport Planning Ltd, 2010; Lucas, 2011; Porter et al., 2017), fostered wellbeing (Irlam and Zuidgeest, 2018; Mbara, 2016; Oviedo et al., 2017) and had potential to break cycles of poverty (Lucas, 2011). However, transport costs had to be traded off against basic needs such as food (Abane, 2011; Diaz Olvera et al., 2013; Integrated Transport Planning Ltd, 2010; Janusz et al., 2019; Kamuhanda and Schmidt, 2009; Kumar, 2011; Poku-Boansi and Cobbinah, 2018; Vermeiren et al., 2015) and residents sometimes ‘worked for the transport’, earning just enough to cover their commuting costs (Lucas, 2011).

[229] [106 excl refs]

#### 3.7.3 Theme 3: Paratransit's significance to livelihoods

Regular mobility was an ingrained part of residents’ livelihood strategies and lack of transport interfered with financial security (Alando and Scheiner, 2016; Andreasen and Møller-Jensen, 2017; Diaz Olvera et al., 2010, 2013; Evans et al., 2018; Janusz et al., 2019; Kamuhanda and Schmidt, 2009; Lucas, 2011; Vermeiren et al., 2015; Yankson et al., 2017). In settings where entrepreneurial activities were high and mode choices were low paratransit was a vital source of mobility, helping residents sustain dense networks and engage in income-generation (Abane, 2011; Evans et al., 2018; Lesteven and Boutueil, 2018; Poku-Boansi and Cobbinah, 2018; Turner and Adzigbey, 2012; Yankson et al., 2017). Paratransit was the powerhouse of the city (Evans et al., 2018), giving economic access to residents in far-flung peripheral areas which were not reached by formal public transport (Andreasen and Møller-Jensen, 2017; Evans et al., 2018; Kamuhanda and Schmidt, 2009; Kola et al., 2012; Kumar, 2011; Lucas, 2011). All but the richest residents used paratransit at least some of the time (Evans et al., 2018; Raynor, 2014) as it allowed for cost-effective commuting to and from formal jobs (Kamuhanda and Schmidt, 2009) and in addition to moving people it provided goods transport, courier services and competitive on-time deliveries (Diaz Olvera et al., 2010; Evans et al., 2018; Kumar, 2011; Raynor, 2014). The paratransit sector in itself was a source of significant jobs for drivers and support workers (eg: those offering repairs or vehicle washing) (Evans et al., 2018).

#### 3.7.4 Theme 4: Paratransit's dual nature

Paratransit was described as having both strengths and problem areas.

***Strengths.*** Paratransit services were ‘gap-fillers’ – ubiquitous, able to penetrate areas where no other transport could and adapted to the landscape, road deficiencies and local weather – and allowed residents to avoid walking long distances (Diaz Olvera et al., 2010, 2013; Evans et al., 2018; Kamuhanda and Schmidt, 2009; Kola et al., 2012; Kumar, 2011; Lesteven and Boutueil, 2018; Lucas, 2011; Poku-Boansi and Cobbinah, 2018; Porter et al., 2017; Raynor, 2014). Services were flexible, with no schedules or fixed stopping points, routes could be varied in real time and services could be individualised (offered on a door-to-door basis, used to transport goods as well as passengers, summoned by phone or a wave, and available to help in emergencies) (Diaz Olvera et al., 2010; Evans et al., 2018; Kumar, 2011; Porter et al., 2017; Raynor, 2014). Paratransit helped overcome both traffic congestion and poor walkability, with two-wheeled modes offering time savings by rapidly negotiating traffic jams – shortened journey times made it attractive to high-income residents who might otherwise drive private vehicles (Evans et al., 2018; Kumar, 2011; Raynor, 2014). Paratransit offered the broadest, densest, most cost-effective connectivity, receiving the biggest share of most peoples' mobility budgets (Kamuhanda and Schmidt, 2009), and its diverse types facilitated multi-modal trips (Kola et al., 2012). One-passenger modes (eg: moto-taxis) could be selected over multi-passenger modes (eg: shared minibus taxis) – valued by women who feared harassment in the latter (Evans et al., 2018; Kumar, 2011). Through its dominance, paratransit was very familiar to residents and strong bonds connected some to their favoured paratransit drivers (Abane, 2011; Evans et al., 2018). Paratransit services extended beyond city boundaries, helping residents maintain connections with home villages and making cities and hinterlands a mobile networked whole (Yankson et al., 2017). The large paratransit sector fitted well into the material and social form of African cities and had some clout as an important source of political support; drivers and users also possessed valuable knowledge which could inform and transform transport strategies (Evans et al., 2018). From a sustainability perspective, paratransit modes like motorcycle taxis were potentially less polluting than private cars (Evans et al., 2018).

***Problem areas****.* Paratransit, as a form of privately owned ‘public transport’, lacked government support and oversight; governance challenges arose from multiple small-scale private operators running many vehicles in flexible ways (Evans et al., 2018). Operating licences were complex and time-consuming to obtain (Mbara, 2016) and no government subsidies were available for vulnerable or needy paratransit users (eg: children, the elderly) (Abane, 2011). Paratransit was costly relative to residents' incomes (Abane, 2011; Diaz Olvera et al., 2013; Evans et al., 2018; Integrated Transport Planning Ltd, 2010; Kumar, 2011; Lucas, 2011; Poku-Boansi and Cobbinah, 2018; Vermeiren et al., 2015), especially for the very poor who needed it most (Janusz et al., 2019), and mode competition and monopolizing by dominant paratransit types affected fare-setting and customer service (Abane, 2011). Over-flexibility resulted in unclear routes, absence of predictable timetables, fare insecurity and at times cheating of users, who were highly vulnerable to service changes like increased fares and decreased availability or speed (Abane, 2011; Kamuhanda and Schmidt, 2009). Users sometimes had to walk long distances due to paratransit services stopping short of key destinations like formal transport hubs, not penetrating certain areas at all (eg: due to poor roads) or being banned from central business districts (Diaz Olvera et al., 2010; Evans et al., 2018; Lucas, 2011; Turner and Adzigbey, 2012). Services were strongly shaped by financial considerations – pressure on drivers to make money for owners caused them to break road rules and inadequately serve less lucrative routes (eg: in far-flung poorer neighbourhoods) (Abane, 2011; Kola et al., 2012; Raynor, 2014). Imbalance between supply and demand led to over-demand in peak periods, with long queues and overloading (in-vehicle congestion) (Kola et al., 2012; Yankson et al., 2017); by contrast, night-time and odd-hours services were limited or non-existent (Andreasen and Møller-Jensen, 2017; Oviedo et al., 2017). Waiting times due to the fill-and-go system were a serious issue, not well captured by researchers – transport modelling underplayed this aspect of accessibility; in addition, travel research tools from high income countries (eg: least-cost path algorithms) failed to reflect how residents' actual travel routes were influenced by practicalities and personal preferences (Andreasen and Møller-Jensen, 2017). It was unclear whether land-use and transport processes were as strongly connected in African cities as in high-income countries (Poku-Boansi and Cobbinah, 2018). Residents reported that paratransit was associated with risks like crime, harassment of women and exposure to the weather (Mbara, 2016; Oviedo et al., 2017; Porter et al., 2017; Raynor, 2014) as well as road crash risks associated with old or poorly maintained vehicles, unfavourable road conditions, dangerous driving, driver fatigue and lack of safety devices for passengers (Kola et al., 2012; Kumar, 2011; Lucas, 2011; Mbara, 2016; Oviedo et al., 2017; Poku-Boansi and Cobbinah, 2018; Porter et al., 2017; Raynor, 2014); some residents addressed safety concerns by praying before journeys (Abane, 2011). Instances of conflict and misbehaviour included disrespect towards passengers by paratransit drivers and conductors (Kamuhanda and Schmidt, 2009) as well as passengers cheating on fare payments, stealing money (or even vehicles), assaulting drivers and pressurizing them to take risks and break rules (Raynor, 2014). Paratransit operators and drivers faced hostile working conditions (“infrastructural violence”) due to unsympathetic government policies and the unsuitable nature of the built environment (Evans et al., 2018; Kola et al., 2012).

#### 3.7.5 Theme 5: The modernist agenda

African cities faced pressures related to modernist thinking about the ‘ideal modern city’, with a focus on aesthetically attractive transport systems, streets serving mainly as corridors for motorized traffic, and planning and development being increasingly car-centric (Alando and Scheiner, 2016; Evans et al., 2018). Road networks were not designed with paratransit in mind (Kola et al., 2012) and in some cities the ‘new economies’ (gas, oil, rubber) had altered residents' financial means and travel patterns, leaving older industries like fishing more marginal and precarious (Yankson et al., 2017). Neoliberal policies and investment focused on lesser-used modes like formal buses and trains (Alando and Scheiner, 2016), with high-profile public transport projects reflecting international trends towards symbolic mega-infrastructure (Evans et al., 2018) and foreign-funded systems like French- and Chinese-funded BRT and light rail (Lesteven and Boutueil, 2018). Nonmotorized modes like walking and cycling were virtually invisible, being devalued and stigmatized as counter to ‘modernity’ (Alando and Scheiner, 2016). Cities' policies disfavoured the poorest groups, inadequately recognizing residents' diverse situations and mobility constraints and prioritizing tourists and middle-class residents over improving mobility for all (Janusz et al., 2019). The conceptual link between transport and wellbeing was overlooked (Oviedo et al., 2017) and policies were mostly aimed at stimulating economic growth rather than social inclusion and equity (Alando and Scheiner, 2016); overly technocratic transport projects resulted in missed development opportunities (Janusz et al., 2019). Some cities overtly legislated against paratransit modes (Evans et al., 2018) and the informal sector was excluded from formal planning discourse (Lesteven and Boutueil, 2018). Many cities expected residents to mode-shift to formal public transport (Abane, 2011) and only some attempted to incorporate paratransit into the system (eg: as a BRT feeder service) (Lesteven and Boutueil, 2018). Legislative bottlenecks were prevalent and a policy impasse or vacuum was present in some cases, with no clear overall responsibility for transport provision and uncoordinated land use and transport planning (Mbara, 2016; Poku-Boansi and Cobbinah, 2018).

#### 3.7.6 Theme 6: Challenging the notion of rogue urbanism

The notion of rogue urbanism was critiqued against the backdrop of general dysfunctionality in African cities' mobility systems (Andreasen and Møller-Jensen, 2017), with an alternative discourse of sustainability offsetting a focus on emulating cities in high-income countries (Evans et al., 2018). The modernist agenda was noted to render transport disadvantage invisible, leading to car-centred development and a failure to allocate space to other modes or allow for multi-modal mobility (Alando and Scheiner, 2016). Transport was increasingly visible as a 21st century city issue, with recognition of the need to shift away from automobility as African cities' populations increased (Evans et al., 2018). Due to limited urban resources it was necessary to build on ‘actually existing’ homegrown solutions which offered social, economic and environmental benefits, rather than on major new investments (Evans et al., 2018; Janusz et al., 2019). Albeit informal, paratransit was noted to be less ‘makeshift’ than other areas of informality like housing and sanitation; the notion of paratransit driving being a transitory occupation was contested as the industry matured (Evans et al., 2018). Paratransit was able to transcend the boundaries between slums and the mega-city, interweaving the formal and informal (Evans et al., 2018); through offering widespread, ubiquitous and affordable connectivity it had become the first choice for many residents, allowing them to escape lengthy, arduous and dangerous trips on foot through hostile environments (Diaz Olvera et al., 2013; Kamuhanda and Schmidt, 2009). It played a major role in everyday mobility, powering businesses and livelihoods (Evans et al., 2018; Lesteven and Boutueil, 2018), and as an emergent capacity it made cities permeable and predictable (Evans et al., 2018). Its existence reduced exclusion and transport disadvantage (Alando and Scheiner, 2016), compensating for the collapse of other modes like rail and water-based transport (Poku-Boansi and Cobbinah, 2018), and its socio-technical capacities helped buffer residents against unpredictable events, enhancing their resilience (Evans et al., 2018). A mix of public transport (including paratransit), walking and private cars was seen as best supporting the needs of different residents, with major projects such as BRT risking overlooking socioeconomic heterogeneity and focusing on major axes and hub-and-spoke patterns with neglect of non-radial routes and polycentric city structures (Janusz et al., 2019; Lesteven and Boutueil, 2018; Vermeiren et al., 2015). In some cities hybrid systems formalised and integrated paratransit modes with formal public transport, extending the reach of the latter by connecting poorer residents to it (Lesteven and Boutueil, 2018; Vermeiren et al., 2015). Technology like the SafeBoda travel-planning app showed potential to organise informal transport modes and erode the boundaries between them and formal modes (Evans et al., 2018). Anti-paratransit city policies represented a form of infrastructural violence, compounding hostile working conditions for paratransit operators (Evans et al., 2018), and the modernist agenda was noted to undermine realistic solutions, failing to recognize the centrality of movement to livelihoods, upwards residential mobility and escaping of cycles of poverty (Alando and Scheiner, 2016; Vermeiren et al., 2015; Yankson et al., 2017). Paratransit associations held some political power but their bottom-up informality left them disconnected from top-down planning and regulatory institutions; improved paratransit legibility by geographers was needed to develop a narrative of legitimacy (Evans et al., 2018).

## 4 Integrated discussion of results

Our review of 41 studies dealing with active travel and paratransit use in African cities showed good coherence between quantitative and qualitative data, suggesting that the ‘lay of the land’ revealed by our quantitative synthesis was well explained by the themes emerging from our meta-ethnography. The studies, in aggregate, elucidated how paratransit facilitated mobility and access to opportunities, on one hand, and was of potential public health benefit – as a key active travel mode – on the other. The first of these two issues received much greater coverage, with the second appearing in the studies more obliquely. Collectively the studies revealed a tension between benefits and harms related to paratransit use. Due to our exclusion of road traffic injuries as well as of sole-purpose travel – for instance, to access health care – we make no claim that our review allows a robust assessment of the relative “sizes” of these benefits and harms.

The reviewed literature did not focus on health as either a determinant or an outcome of paratransit use. Whilst some studies mentioned safety this was as a general travel concern and did not influence residents' choices to use or not use paratransit. Nor were safety problems reported as a unique outcome of paratransit travel, as compared to other modes. It was apparent that paratransit use intersected with two other forms of active travel (walking and use of formal public transport) but had little or no intersection with cycling, possibly due to cycling's low mode share in most of the cities. Paratransit also intersected with non-active travel (private car and motorbike use), at least as a back-up option, suggesting potential to combat broader-scale mode-shifting to private vehicles.

With respect to how residents chose between and experienced different travel modes, choices were shaped particularly by income-poverty and access-poverty, with poorer residents in peripheral areas having high mobility needs but very constrained choices. Travel realities in African cities included time-poverty, with females having time pressure due to household responsibilities and peripheral residents losing time to long, slow journeys. Traffic congestion (a dominant feature) also created time-poverty for private car owners, some switching to two-wheeler paratransit for faster travel. Energy-poverty was experienced by residents who lived long distances from their destinations, faced hostile walking terrain or needed to transport

heavy goods; access to paratransit improved the situation by allowing for less onerous active travel. Safety-poverty was reported by residents as a feature of travel in general and did not strongly influence mode choice, despite some concerns about paratransit safety. Finally, only wealthier residents could select modes based on factors such as comfort and convenience. Taken as a whole, the studies reflected that whilst paratransit is of overwhelming importance for mobility it is at the same time not sufficient to ensure equity and optimal accessibility. This dovetails well with the recent findings of Massingue and Oviedo in Mozambique (Massingue and Oviedo, 2021) and those of Nakamura and Avner in Kenya (Nakamura and Avner, 2021). The former noted, in setting the context for their walking-focused research (framed by the Right to The City or RTTC), that the Maputo metropolitan area was plentifully supplied with both formal and informal public transport, but residents in numerous areas faced cost barriers to paratransit use. The latter noted that in Nairobi only 10% of jobs were reachable within an hour of walking and only 25% were accessible via paratransit in the form of minibus taxis.

An important finding of our review was the uneven geographical representation in the literature, omitting countries and entire regions; many countries failed to show up at all in the data whilst others appeared multiple times. Central Africa and Northern Africa were significantly under-represented, while disproportionate data was present in relation to Ghana and Nigeria (Western Africa), Kenya and Uganda (Eastern Africa) and South Africa (the only Southern African country represented). In terms of the state of the literature – at least during the period under review – we found that study quality was difficult to judge, mainly because of under-reporting in relation to ethical and methodological issues including data analysis techniques. We found that modified CASP checklists were imperfectly suited to rating study quality; in particular, many quantitative studies were ‘penalised’ for their reliance on self-reported data, despite the fact that this is commonly used in travel behaviour research especially in under-resourced settings. We cannot readily comment on how the quality issues we identified compare with quality issues which may be found in bodies of literature in other fields or disciplines, or comparable sets of travel behaviour studies from other geographical regions – to do so would require a study in itself. Similarly, we cannot comment on whether the high prevalence of out-of-country authors we noted is unusual, or is also apparent in relation to travel behaviour research in other world regions. Given the high proportion of studies with out-of-country senior author affiliations and foreign funding, we note the importance of ensuring that researchers in low- and middle-income countries are equitable partners in generating and sharing knowledge. High-income country researchers need to be sensitive, strive to avoid tokenism and extractive practices and transparently provide information as to how language and cultural differences were negotiated during the research process (Armenteras, 2021).

With regards to the reproducibility of our research, by providing a detailed account of our methodology we hope to have created a road-map which can be used for a similar review of the literature from other world regions in which paratransit forms part of the transport mix, such as South East Asia, or from other time periods. With regards to whether our precise findings would be reproduced by a different research team working through the same set of studies, this may of course depend on the particular make-up of the team. Our team contained a preponderance of health disciplines whilst another team with a stronger transport, human geography or sociological lens may have interpreted the findings of the studies somewhat differently. As described earlier, we did seek to create balance by testing our emerging findings against key informants from the transport sector as well as transport-focused experts from the health sector. Future research of benefit to public health could include better quantification of the extent to which physical activity is associated with paratransit use, in particular cities or across cities. This could, for instance, involve measuring distances walked by residents to access paratransit and at journey's end when walking to their destinations. Further multidisciplinary studies could use both a public health lens and a sustainability/planetary health lens to more definitively explore the degree to which paratransit availability offsets mode-shifting to private cars. Transport- and policy-oriented researchers, by being sensitive to issues of public health, could usefully analyse the tension between harms and benefits in settings with anti-paratransit policies – in particular, whether such policies have unintended health outcomes like reducing active travel or preventing access to health care.

Of note for policymakers and transport practitioners, the studies overall made it clear that African city residents in the represented countries, faced with dysfunctional mobility systems and a mobility gap, had actively embraced paratransit as an effective connector to key destinations, especially in relation to livelihood-generation. This was particularly true for the peripheral poor and in cities where colonial or apartheid transport systems had collapsed or ‘new economies’ had altered travel patterns, increasing the inadequacy of formal public transport. Paratransit's ubiquity and informality were strengths, as experienced by users, and fostered its proliferation even given its problematic elements –, entrenching it in the travel behaviour of many residents. Privately owned, profit-driven small-scale paratransit operations had competitive advantages when it came to providing services in informal, unplanned areas with poor road networks, inaccessible to other modes and typically under-served by formal public transport. Thus, the footprint of paratransit in African cities significantly exceeded that of formal modes like buses, constrained by formal schedules and designated routes and stopping places, as well as the existing or potential footprints of new systems like BRT which risked being out of reach of poorer residents, financially and spatially. The reviewed literature showed that paratransit had proliferated despite hostile working environments characterized by disapproval from city authorities with a modernist agenda, dislike of ‘rogue urbanism’ and general disregard for the informal sector, and despite governments' tendency to focus on car-centric road-building projects. Apart from the very poor (who could not afford any fares and remained captive walkers) and residents whose neighbourhoods were not penetrated by *any* transport, paratransit served residents well and helped prevent complete immobility or confinement to virtual travel (eg: by mobile phone). It facilitated common travel behaviours like multi-modal journeys, including switching between paratransit types, like motorbike taxis and minibus taxis. It co-existed with and – when means allowed – substituted for the dominant mode of walking and, by providing feeder and first-mile/last-mile services, it improved access to formal public transport. It was also in some demand from vehicle-owners as a back-up and at times a preferential option.

We noted contradictions between city authorities’ views of what mobility *should* look like (from a modernist perspective) and what mobility *did* look like in African cities. Despite road-building projects (government- or foreign-funded) and introduction of new formal public transport modes like BRT, neoliberal resource constraints created dysfunctional urban mobility systems, especially in smaller cities. Despite its problems, paratransit showed itself to be an already-existing, locally adapted, indigenous solution and a relatively affordable and efficient mode. We noted its potential to minimise active travel harms by replacing onerously long foot trips as well as arduous load-carrying for all but the poorest. To help realise its full potential to address the mobility gap and maximise the benefits of active travel African city authorities may need to address financial barriers to its use. Addressing road network factors by designing roads suited to paratransit operations could help ensure reasonable access in more parts of the city. Where the majority of city residents are not served (or not likely to be served) by formal systems like BRT, there is a risk that government and foreign investment will serve the elite and worsen equity gaps, whereas focusing on effective mobility for the population at large would favour those at greatest disadvantage (eg: females and poor residents in informal peripheral settlements).

Arguably, paratransit operations are not truly rogue in the sense of being “*not expected* or *not normal*”^7^; instead, they show logical demand-responsive proliferation patterns and have evolved clear rules of operation (like the fill-and-go system) which are familiar to and understood by residents. Bolstering rather than reducing paratransit's attractiveness can mitigate practical and aspirational reasons for rising private vehicle ownership, enhancing cities' long-term sustainability and planetary health. With its wide footprint, versatility and homegrown quality, paratransit has a particular role to play as it “actively interweave[s] informal and formal elements of the city” (Evans et al., 2018, p. 682); hybrid integrated public transport systems, with seamless integration between paratransit and other modes, appear best placed to ensure mobility for all city residents.. Political will is required to reshape the built environment and policy context to be less hostile towards and neglectful of paratransit operators and users, whilst addressing roguish elements. Whilst our review did not delve in depth into these elements, they were seen to include personal security and road safety risks, violent intra-mode competition, inefficiencies, pollution by older or defective vehicles, fare instability and variable customer service levels.

Like us, other researchers have noted that informal collective transport is dominant across the African continent, accounting for over a third of motorized urban travel (Behrens et al., 2016; Bogale, 2012). Some have made a similar case for improving public transport in African cities by favouring hybrid systems in which demand-responsive, innovative paratransit forms part of the officially recognized mode mix (Behrens et al., 2016). Some have recommended adoption of a rights-based approach (‘the right to the city’) in which public transport enables the realisation of political, civil and socioeconomic rights (Coggin and Pieterse, 2015). Finally, other researchers have noted that few African countries have active travel policies, a gap which needs to be remedied as noncommunicable diseases become more prevalent (Loo and Siiba, 2019).

Our review's limitations stemmed partly from heterogeneity in the studies, making it difficult to compare their findings and requiring the use of bespoke methods, as well as the aforementioned geographic gaps in the data. Although we offer a rich synthesis, given this unevenness and the diversity of African countries (with their unique histories and topographical and sociocultural realities) we make no claims that our findings can be generalised to the continent as a whole. Furthermore, the quality of the studies was uneven with special concerns arising from lack of evidence of researcher reflexivity and limited information on how language and cultural differences between out-of-country researchers and participants were handled. The review's strengths include that it is the first to draw together mixed-method research (including meta-ethnography) into active travel and paratransit in African cities, effectively critiquing the somewhat simplistic narrative around active travel and public transport being unequivocally desirable which runs through a great deal of the transport and health literature from high-income countries. Through adding a meta-ethnography we also went beyond a simple summary of the heterogeneous evidence and applied rigorous and established interpretive strategies to achieve a more meaningful overarching summation. Rigour was enhanced by our large research team, encompassing different disciplines and geographic origins. Our 19-strong author group includes 10 (53%) with African affiliations – one each in Cameroon (Western Africa) and Kenya (Eastern Africa) and eight in South Africa (Southern Africa). Furthermore, discussion with key informants from international organisations lent depth and nuance to our analysis, complementing the thickness derived from our reflexive notes. All in all, the review is a rich and trustworthy synthesis of a large set of mixed-method data.

## 5 Conclusions

Our review of the literature expands the traditional understanding of public transport as an active travel mode by focusing specifically on research into paratransit use in urban African settings. This is of importance given the epidemiological transition underway on the continent, with noncommunicable diseases rising as populations urbanise and motorization increases. The reviewed studies showed that paratransit, a key part of the public transport mix in African cities, helped meet residents' diverse travel needs whilst, as a form of active travel, contributing to public health by offsetting noncommunicable disease risks. It could substitute for onerously long foot journeys and manual load-carrying and expanded residents’ access to formal public transport. It also showed promise in relation to reducing unsustainable mode-shifting to private vehicles. The infrastructural violence and policy hostility towards paratransit which were apparent in the literature seem misplaced and shortsighted. As an already-existing and much-used active travel mode, we conclude that paratransit deserves prioritizing over elite-serving formal public transport projects. Formalising of its various types could be helpful but we suggest that this should not be at the expense of its agility, demand-responsiveness and ability to penetrate under-served poor and peripheral neighbourhoods. We recommend that mechanisms be sought to improve its affordability for the poor and to address its personal security and road safety challenges.

A public health lens can and should be applied to travel and transport research, whilst public health researchers should acquaint themselves with evidence from the transport and social science disciplines. Through multidisciplinarity and the addition of methods like meta-ethnography to systematic reviews, it is possible to converge on conclusions which are critical, nuanced and go beyond a mere summation of the extent literature. Future research of the types outlined, using a health lens and innovative methodologies and tools, can make paratransit – and residents' active travel behaviour in general – more legible to city authorities. This will help foster sustainable mobility systems which offer an optimal traffic mix to meet residents’ needs, whilst preserving active travel as a public health measure in a rapidly urbanising Africa.

## Funding

This work was supported by the National Institute for Health Research (NIHR) (GHR: 16/137/34) using UK aid from the 10.13039/100013986UK Government to support global health research. The views expressed in this publication are those of the authors and not necessarily those of the NIHR or the UK Department of Health and Social Care.

Lee Randall and Safura Abdool Karim were supported by the SAMRC/Wits Centre for Health Economics and Decision Science-PRICELESS-SA, School of Public Health, University of the Witwatersrand (SAMRC grant/project code 23108). Anna Brugulat-Panés was funded by the UKRI Economic Social Research Council Doctoral Training Program (ESRC DTP) and by the MRC Epidemiology Unit, 10.13039/501100007552School of Clinical Medicine, University of Cambridge. Lambed Tatah was funded by a Cambridge International Islamic Development Bank PhD scholarship. Gabriel Okello held an Air Quality and NCDs fellowship supported by a philanthropic gift from AstraZeneca, grant number G111521.

No funders played a role in study design, data collection and analysis, decision to publish, or preparation of the manuscript.

## Author contribution statement (CRediT model)

**Conceptualization**: LR, LF, ABP, JW, LJW, LM, LT, IH, FA. Funding **acquisition**: JW, LJW, LM, IH, FA. **Data curation**: LR, LF, ABP, JW, LKW, CP, LM, GM, LT, PMD, SSM, IH, GO, MA, HK, ACA, GPBG. **Methodology**: LR, LF, ABP, IH. **Supervision**: LR, LF, ABP, JW, LJW, LM. **Formal analysis**: LR, LF, ABP, JW, LJW, CP, SAK, LM, GM, LT, PMD, GO, FA. **Investigation**: LR, LF, ABP, JW, LJW, CP, LM, GM, LT, PBD, SSM, IH, GO, MA, HK, ACA, GPBG. **Validation**: LR, LF, ABP, JW, SAK. **Project administration, resources, visualisation**: LR, LF, ABP. **Writing (original draft)**: LR, LF. **Writing (review & editing)**: all authors.

## Declaration of competing interest

The authors declare no competing interests.

## Appendix A Supplementary data

Multimedia component 3 Multimedia component 3

Supplementary Table 1 Paratransit types reflected in the studies, with study regions shown. Supplementary Table 1

## Footnotes

1 https://www.crd.york.ac.uk/prospero/display_record.php?RecordID=124802.

2 https://www.covidence.org.

3 https://trello.com.

4 https://casp-uk.net.

5 https://jamboard.google.com.

6 www.gdarnet.org.

7 Cambridge Dictionary of English.
