# Supplementary material for: Active travel and paratransit use in African cities: Mixed-method systematic review and meta-ethnography
Source: J Transp Health. 2023 Jan;28:101558. doi: 10.1016/j.jth.2022.101558 (PMC9902334; doi:10.1016/j.jth.2022.101558)
Supplement: Multimedia component 1 [file mmc1.docx]

**Supplementary File 2: Data extraction template**

| **Field** | **Standard or custom  Covidence field** | **Format** | **Notes** |
| --- | --- | --- | --- |
| **Identification** |  |  |  |
| Study details |  |  |  |
| Sponsorship source | Standard | Free text | Check whether a funding source is reported. Often this can be found in a section titled ‘Acknowledgements’ or ‘Funding’ or reported on the first page of the manuscript near the author information.  Enter the funding source  If more than one, list separated by a comma  Otherwise enter ‘no source reported’  *Example*  Medical Research Council, Heart Foundation |
| Country | Standard | Free text | Enter the country where the data were collected  If more than one, list separated by a comma  *Example*  Ghana |
| Setting | Standard | Free text | Enter the setting where the data were collected  If more than one, list separated by a comma  If information given at different levels of geographical aggregation (e.g. region, city, district), list all this information and where possible give information on the level of aggregation in brackets  *Example*  Accra, Kumasi, Tamale, Sekondi–Takoradi (metropolitan areas) |
| Comments | Standard | Free text | Leave this field blank or use for general comments/queries arising |
| Author’s contact details |  |  |  |
| Author's name | Standard | Free text | Enter the name of the first author  Use the format [surname, initials]  *Example*  Abane, AB |
| Institution | Standard | Free text | Enter the institution or affiliation of the first author with the country of this affiliation in brackets  If more than one, list separated by a comma  *Example*  University of Cape Coast (Ghana) |
| Email | Standard | Free text | Enter the email address of the first author  If none provided enter ‘email not provided’  *Example*  [am_abane@yahoo.com](mailto:am_abane@yahoo.com) |
| Address | Standard | Free text | Leave this field blank |
| Additional identification data |  |  |  |
| Country - first author | Custom | Free text | Enter the country where the first author’s institution or affiliation is located  If more than one, list the country of the first affiliation  *Example*  Kenya |
| Country - last author | Custom | Free text | Enter the country where the last author’s institution or affiliation is located  If more than one, list the country of the first affiliation  If the study has only one author, enter ‘no last author’  *Example*  United States of America |
| Data source | Custom | Free text  Categorical | Enter the source of the data  Use the following categories:  If more than one, list separated by a comma   - Academic - Research Institute/Centre - Government - Private sector - International organisation - NGO - Other   Academic: research studies led by universities  Research Institute/Centre: research studies led by research groups which are part of a larger institution such as universities, hospitals, corporations, etc.  Government: routine monitoring such as national surveys or traffic monitoring  Private sector: research studies led by industry, private companies, consultancy firms, etc.  International organisation: research studies led by organisations such as WHO, World Bank, FAO, UN, etc.  NGO: research studies led by non-profit, non-governmental organisations  Other: enter other type of source of data not listed above  *Example*  Academic, Research Institute/Centre, International organisation |
| Date - data collection | Custom | Free text | Enter the start and end year of data collection  If data collection was completed within one year, enter the year only  If one discrete period of data collection spanned multiple years, enter the start and end year separated by a dash  If there were multiple discrete periods of data collection (e.g. in a longitudinal cohort that had a baseline and follow-up data collection), enter the years separated by a comma  *Examples*  2014  2014-2015 (one discrete period of data collection)  2014, 2017 (multiple periods of data collection e.g. cohort)  2015-2015, 2017-2018 (multiple periods of data collection, each spanning multiple years) |
| Date - publication | Custom | Free text | Enter the year that the study was published  *Example*  2014 |
| GDAR - work package 1b | Custom | Free text | Enter ‘WP1flag’ if the study uses spatial assessment, or provides information on transportation patterns, physical activity patterns, road traffic accidents, air pollution, demographics or health outcomes  Otherwise enter ‘none’ |
| GDAR - work package 3 | Custom | Free text | Enter ‘WP3flag’ if the study refers to policy in South Africa, Kenya, Cameroon or Jamaica, and provides information on the sector of origin of the policy such as health or transport  Otherwise enter ‘none’ |
| Equity | Custom | Free text | Enter ‘equityflag’ if the study explores gender or sex differences in travel behaviour, or differences by socioeconomic status to inform an equity analysis  Otherwise enter ‘none’ |
| **Methods** |  |  |  |
| Design |  |  | Leave this field blank |
| Additional methods data |  |  |  |
| A. Research type | Standard | Categorical | Enter the research type:  Use ONE of the following categories:   - Quantitative - Qualitative - Mixed method   Quantitative: measures of values or counts expressed as numbers e.g. the data generated from surveys, questionnaires, measurements  Qualitative: description of phenomena often expressed as text e.g. interviews, focus groups  Mixed method: Both quantitative and qualitative methods used |
| B. Study design - quantitative | Custom | Free text  Categorical | Enter the study design.  Quantitative or mixed method study - use ONE of the following categories:   - Randomised controlled trial - Cohort study - Case-control study - Cross sectional study - Case report / Case study - Other – please, specify   For quantitative or mixed method studies, complete this field. Otherwise (i.e. for qualitative studies) enter ‘not applicable’ |
| C. Study design - qualitative | Custom | Free text  Categorical | Enter the study design.  Qualitative or mixed method study – use ONE of the following categories:   - Grounded theory - Case study - Historical / Narratives - Participatory research / Action research - Phenomenology - Ethnography / Observation - Other – please, specify   Ethnography: Immersion of the researcher the participants’ environment, typically through observation (note, do not confuse this design with observational quantitative designs like traffic counting)  For qualitative or mixed method studies, complete this field. Otherwise (i.e. for quantitative studies) enter ‘not applicable’ |
| D. Study method – quantitative | Custom | Free text | If the study includes a quantitative element, enter the method type.  Enter a small amount of free text summarising the study methods. This can usually be cut and pasted from the abstract.  *Examples*  Two-day vox pop survey, structured questionnaire at two main trip destinations  STEPs survey  Household survey and interview  For quantitative or mixed method studies, complete this field. Otherwise (i.e. for qualitative studies) enter ‘not applicable’ |
| E. Study method - qualitative | Custom | Free text  Categorical | If the study includes a qualitative element, enter the method type.  Use the following categories:  If more than one, list separated by a comma   - Structured – interview, survey, questionnaire - Un-structured or Semi structured - interview, survey, questionnaire - In depth- interview / Key informants - Focus groups / Group discussions - Field Notes - Narrative descriptions - Audio tapes - Video tapes - Seasonal calendars - Transect walks - Participatory mapping / Modelling - Other– please, enter a small amount of free text summarising the study methods.   For qualitative or mixed method studies, complete this field. Otherwise (i.e. for quantitative studies) enter ‘not applicable’ |
| F. Exposure category | Custom | Free text  Categorical | Enter ALL of the categories of exposure that were assessed in the study.  Use the following categories:  If more than one, list separated by a comma   - Individual characteristics - Travel mode characteristics - Built environment - Natural environment - Socio-cultural environment - Policy or wider environment   See the ‘Categories of exposure’ (Table 1) in this document to help you decide which exposures should be listed.  Exposures must be linked explicitly with travel behaviour outcomes, not just reported. E.g. A study might report the sex distribution of the sample (i.e. numbers of males and females), but to be considered an exposure the study needs to have examined how travel behaviour varies by sex (e.g. whether public transport use differs between males and females). For this example, the following category should be entered: ‘Individual characteristics’. |
| G. Exposure/s | Custom | Free text | Enter all of the exposures that were assessed in the study. See Table 1 to help you decide how to report exposures. |
| H. Exposure methods | Custom | Free text  Categorical | Enter ONE of the methods of exposure that were assessed in the study:   - Subjective - Objective - Both subjective and objective   Subjective: Usually this means the participant has self-reported  Objective: Observed by the researcher. For example, measured population density using census data, measured land use mix using geographical information systems |
| I. Outcome/s | Custom | Free text  Categorical | Enter ALL of the following outcomes that were assessed in the study:  Enter all outcomes assessed from this list, separated by a comma.   - Time in all travel - Time in active travel - Time in travel modes or combinations of modes - Number of trips/journeys - Use of travel modes - Choice of travel modes - Travel mode share - Mobility budget (percentage) - Purpose of travel mode use - Other – please, specify   Active travel is commonly operationalised as walking only, cycling only, walking and cycling combined, or walking, cycling and public transport combined  Enter all outcomes assessed from this list, separated by a comma  *Example*  Time in all travel, time in active travel |
| J. Outcome methods | Custom | Free text  Categorical | Enter ONE of the methods of outcome that were assessed in the study:   - Subjective - Objective - Both subjective and objective   Subjective: Usually this means the participant has self-reported  Objective: Observed by the researcher. For example, counting people using particular travel modes |
| K. Analysis method - quantitative | Custom | Free text | Enter a small amount of free text summarising the analysis methods. For quantitative studies it is important to distinguish between basic statistical methods for assessing relationships (e.g. correlations) and those that account for potential confounding factors (e.g. multivariable regression modelling).  For quantitative or mixed method studies, complete this field. Otherwise (i.e. for qualitative studies) enter ‘not applicable’ |
| L. Analysis method - qualitative | Custom | Free text | Enter a small amount of free text summarising the analysis methods, e.g. thematic analysis.  For qualitative or mixed method studies, complete this field. Otherwise (i.e. for quantitative studies) enter ‘not applicable’ |
| **Population** |  |  |  |
| Inclusion criteria | Standard | Free text | Enter a small amount of free text summarising the types of participants included in the study. This can usually be cut and pasted from the article.  *Example*  Data were collected from passengers waiting to board or actually on board vehicles operated by privately-owned and government-assisted transport systems in the four metropolitan areas under study. |
| Exclusion criteria | Standard | Free text | Enter a small amount of free text summarising the types of participants excluded from the study. This can usually be cut and pasted from the article.  In many cases, there will be no explicit exclusion criteria listed. If this is the case, enter ‘not reported’.  *Example*  Institutionalised individuals (e.g. hospitalised or jailed) were excluded from the study  Participants with a diagnosis of cancer were excluded |
| Group differences | Standard | Free text | Enter a small amount of free text summarising whether there were any differences between participant groups at baseline. This only applies to studies that used a controlled experimental design (and it is likely that the majority of literature identified will not use this type of design). This can usually be cut and pasted from the article.  If this does not apply, enter ‘not applicable’. |
| Additional population data |  |  |  |
| 1. Sample size | Custom | Free text | Enter a small amount of free text describing the sample size  *Examples*  10,128 individuals  100 households, 1 member of each household  Ouagadougou - 754 households, 3682 individuals, Bamako - 251 households, 1666 individuals |
| 2. Response rate | Custom | Free text | Enter a small amount of free text describing the response rate, if provided. If reported, this is typically expressed as a percentage. It is likely that many studies will not provide this information.  If not reported, enter ‘not reported’  *Example*  78% response rate |
| 3. Age | Custom | Free text | Enter a small amount of free text describing the age of participants. If possible, enter this in the format ‘mean (standard deviation)’. Other common reporting formats are a range, or the proportion of participants that fall within a particular age range.  If not reported, enter ‘not reported’  *Examples*  45.3 (2.1) years  15-60 years  48% of participants were aged 30-44 years  Over 13 years |
| 4. Sex | Custom | Free text | Enter a small amount of free text describing the sex of participants. If possible, enter this in the form ‘number (percentage)’  If not reported, enter ‘not reported’  *Examples*  100 (25%) female; 300 (75%) male  55% female  Female only |
| 5. Urban/rural | Custom | Free text | Enter a small amount of free text describing the urban/rural status of participants  If not reported, enter ‘not reported’  *Examples*  Rural  Urban  Urban and peri-urban dwellers |
| 6. Direction of relationships | Custom | Free text | Enter a small amount of free text summarising the direction of the relationships found between exposures and outcomes. Try to keep this as succinct as possible, although it is likely that some studies will report a large number of relationships. Do not worry about reporting the size of the relationship.  *Examples*  Women travelled less than men  Where new roads were built, car traffic increased |
| 7. Main idea | Custom | Free text | Imagine you would like to summarise the article to a colleague in few sentences. What would you tell him/her?  Try to get the main idea of the article. Limit your answer to 1 paragraph containing 3 to 5 sentences (maximum) |
| Baseline characteristics |  |  |  |
| **Interventions** |  |  | Do not enter data in this section |
| **Outcomes** |  |  | Do not enter data in this section |

**Categories of exposure**

| **Exposure category** | **Examples** |
| --- | --- |
| Individual characteristics | Age  Sex  Ethnicity  Urban vs. rural dweller  Slum dweller  Country, region or area of residence  Socioeconomic status  Household factors (e.g. size, assets)  Employment status  Mobility budget (e.g. the share of income spent on public transport)  Education level  Occupation  Income  Marital status  Vehicle ownership (e.g. car or motorbike)  Bike ownership  Hold driving license  Ability to cycle  Attitude towards particular travel modes  Knowledge about travel modes  Distance needed to travel (e.g. to place of work)  Lifestyle  Health status |
| Travel mode characteristics | Safety (e.g. road traffic accidents, injuries or fatalities)  Crime (e.g. interpersonal violence, harassment, mugging)  Cost  Comfort  Customer service  Accessibility  Availability of services  Availability of space  Speed (e.g. traffic jam, delays)  Security  Price stability |
| Built environment | Population density/urban sprawl  Land use mix  Street connectivity  City layout (e.g. shops or other community destinations all clustered in city centre)  Transport infrastructure (e.g. provision of paved roads, public transport, footpaths/cycling paths or infrastructure that protects vulnerable users)  Sidewalk or street-crossing quality  Cycle path quality  Street lighting  Speed management infrastructure  General levels of traffic  Green space  Pollution or air quality  Aesthetics (e.g. pleasantness of area)  Incivilities (e.g. crime, litter, upkeep of area, traffic noise)  Note: This includes individuals’ perceptions of the built environment as well as objective assessments of the built environment |
| Natural environment | Climate (e.g. rainy vs. dry season, winter vs. summer)  Weather (e.g. hot temperatures)  Natural disasters  Note: This includes individuals’ perceptions of the natural environment as well as objective assessments of the natural environment |
| Socio-cultural environment | Social norms (e.g. family/household member roles, or community values)  Gender roles  Religious norms  Cultural norms  Class segregation  Stigmatisation/aspiration of particular travel modes (e.g. car seen as indicator of wealth) |
| Policy or wider environment | Urban planning (e.g. planned vs. unplanned development)  Regional or national travel budgets or policies (including policy priorities)  Regional or national physical activity policies (including policy priorities)  Transport sector regulation/management, including taxes  Corruption/mismanagement  Economic development (e.g. leading to changing types of occupations)  Legacies (e.g. of apartheid or colonisation)  Climate/sustainability agenda (including oil crisis)  Overseas aid  Globalisation  International policy priorities  War and conflict |
