# Supplementary material for: Active travel and paratransit use in African cities: Mixed-method systematic review and meta-ethnography
Source: J Transp Health. 2023 Jan;28:101558. doi: 10.1016/j.jth.2022.101558 (PMC9902334; doi:10.1016/j.jth.2022.101558)
Supplement: Multimedia component 2 [file mmc2.docx]

Supplementary file 3: Details of included studies

| **First author, year** | | **Country/countries (city/cities)** | **African region** | **Research design**  **(Methodology)** | **Author affiliations** | **Funding** | **Paratransit mode(s) mentioned** |
| --- | --- | --- | --- | --- | --- | --- | --- |
| Quantitative studies | | | | | | | |
| 1 | Aworemi, 2008 ([Aworemi et al., 2008](#_ENREF_5)) | Nigeria  (Ilorin) | Western Africa | Quantitative  (cross-sectional survey) | First author: Nigeria  Last author: Nigeria | No funding reported | - Danfo minibus^ii^ - Molue (also known as bolokaja)^iii^ |
| 2 | Behrens, 2010 ([Behrens & Schalekamp, 2010](#_ENREF_8)) | South Africa  (Cape Town) | Southern Africa | Quantitative  (cross-sectional survey) | First author: South Africa  Last author: South Africa | African Centre for Excellence for Studies in Public and Non-motorized Transport, funded by Volvo Research and Educational Foundations; Cape Town Metrorail | - Minibus taxi^ii^ |
| 3 | Elfiky, 2010 ([Elfiky, 2010](#_ENREF_20)) | Egypt  (Kafr Elshiekh) | Northern Africa | Quantitative  (cross-sectional survey) | Single author: Egypt | None reported | - Toc-toc^i^ - Microbus^ii^ - Minibus^ii^ |
| 4 | Salon, 2010 ([Salon & Gulyani, 2010](#_ENREF_66)) | Kenya  (Nairobi) | Eastern Africa | Quantitative  (cross-sectional survey) | First author: United States of America  Last author: United States of America | World Bank; Norwegian Trust Fund | - Matatu^ii^ |
| 5 | Bwire, 2011 ([Bwire, 2011](#_ENREF_11)) | Tanzania  (Dar es Salaam) | Eastern Africa | Quantitative  (cross-sectional survey) | Single author: Tanzania | African Centre for Excellence for Studies in Public and Non-motorized Transport, funded by Volvo Research and Educational Foundations | - Daladala^ii^ |
| 6 | Muhammed, 2011 ([Muhammed, 2011](#_ENREF_50)) | Nigeria  (Kano state) | Western Africa | Quantitative  (cross-sectional survey) | Single author: Nigeria | None reported | - Tricycle^i^ |
| 7 | Mutiso, 2011 ([Mutiso & Behrens, 2011](#_ENREF_51)) | Kenya  (Kisumu and Nakuru) | Eastern Africa | Quantitative  (cross-sectional survey) | First author: South Africa  Last author: South Africa | Mombasa Polytechnic; Volvo Research and Educational Foundations | - Boda boda bicycle taxi^viii^ - Boda boda motorcycle taxi^iv^ - Tuk tuk^i^ - Matatu^ii^ |
| 8 | Venter, 2011 ([Venter & Badenhorst, 2011](#_ENREF_75)) | South Africa  (Gauteng province) | Southern Africa | Quantitative  (repeat cross-sectional survey) | First author: South Africa  Last author: South Africa | Gauteng City Region Observatory funded by University of Johannesburg, University of the Witwatersrand and Gauteng Provincial Government | - Minibus taxi^ii^ |
| 9 | Nwaogbe, 2012 ([Nwaogbe et al., 2012](#_ENREF_53)) | Nigeria  (Aba) | Western Africa | Quantitative (cross-sectional survey) | First author: Nigeria  Last author: Nigeria | No funding reported | - Keke napep (tricycle)^i^ - Okada^iv^ |
| 10 | Salon, 2012 ([Salon & Aligula, 2012](#_ENREF_65)) | Kenya  (Nairobi) | Eastern Africa | Quantitative (cross-sectional survey) | First author: United States of America  Last author: Kenya | Volvo Research and Educational Foundations; The Earth Institute at Columbia University, USA | - Matatu^ii^ |
| 11 | Weinstock, 2015 ([Weinstock et al., 2015](#_ENREF_77)) | Kenya  (Nairobi) | Eastern Africa | Quantitative (cross-sectional survey) | First author: not reported  Last author: not reported | Institute for Transportation and Development Policy (funding from Climate Works) | - Matatu^ii^ |
| 12 | Diaz Olvera, 2016 ([Diaz Olvera et al., 2016](#_ENREF_18)) | Senegal  (Dakar) | Western Africa | Quantitative (repeat cross-sectional survey) | First author: France  Last author: France | No funding reported | - Motorbike taxi^iv^ - Car rapide^ii^ - Ndiaga Ndiaye^ii^ - Clandos (shared taxi)^vi^ - Horse-drawn cart^vii^ |
| 13 | Olawole, 2016 ([Olawole & Olapoju, 2016](#_ENREF_55)) | Nigeria  (Ife-Ife) | Western Africa | Quantitative  (cross-sectional survey) | First author: Nigeria  Last author: Nigeria | No funding reported | - Motorcycle taxi^iv^ |
| 14 | Saddier, 2016 ([Saddier et al., 2016](#_ENREF_64)) | Ghana  (Accra) | Western Africa | Quantitative (GIS analysis) | First author : Ghana  Last author : Canada | Canada Fonds Québécois de la Recherche sur la Société et la Culture Nouveaux Chercheurs Program;  Canada Research Chairs Program;  Canadian Foundation for Innovation | - Jitney (also known as trotro)^ii^ |
| 15 | McKay, 2017 ([McKay et al., 2017](#_ENREF_46)) | South Africa  (Gauteng Province) | Southern Africa | Quantitative (cross-sectional survey) | First author: South Africa  Last author: South Africa | Gauteng City Region Observatory | - Minibus taxi^ii^ |
| 16 | Sabry, 2017 ([Sabry et al., 2017](#_ENREF_63)) | Egypt  (Alexandria) | Northern Africa | Quantitative (cross-sectional survey) | First author: Egypt  Last author: Egypt | No funding reported | - Cycle rickshaw^v^ - Shared three-wheeler taxi ^i^ |
| 17 | Chakwizira, 2018 ([Chakwizira et al., 2018](#_ENREF_12)) | South Africa  (Gauteng Province) | Southern Africa | Quantitative (cross-sectional survey) | First author: South Africa  Last author: Nigeria | No funding reported | - Minibus taxi^ii^ |
| 18 | Scorcia, 2018 ([Scorcia & Munoz-Raskin, 2018](#_ENREF_67)) | South Africa (Johannesburg) | Southern Africa | Quantitative (case study) | First author: United States of America  Last author: United States of America | No funding reported | - Minibus taxi^ii^ |
| 19 | Tembe, 2018 ([Tembe et al., 2018](#_ENREF_68)) | Kenya  (Nairobi)  Mozambique (Maputo) | Eastern Africa  Eastern Africa | Quantitative (cross-sectional survey) | First author: Japan  Last author: Japan | Japan International Cooperation Agency | - Chapas^ii^ - Matatu^ii^ |
| Mixed-method studies | | | | | | | |
| 1 | Diaz Olvera, 2010 ([Diaz Olvera et al., 2010](#_ENREF_19)) | Cameroon  (Douala)  Niger  (Niamey) | Central Africa  Western Africa | Mixed-method (cross-sectional survey, in-depth interviews) | First author: France  Last author: Cameroon | No funding reported | - Bendskin (motorbike taxi)^iv^ - Shared taxi^ii^ |
| 2 | Integrated Transport Planning (ITP), 2010 ([Integrated Transport Planning Ltd., 2010](#_ENREF_30)) | Uganda  (Greater Kampala Metropolitan Area) | Eastern Africa | Mixed-method (cross-sectional survey, traffic counts, semi-structured interviews) | Single author: United Kingdom | No funding reported | - Matatu^ii^ |
| 3 | Abane, 2011 ([Abane, 2011](#_ENREF_1)) | Ghana  (Accra, Kumasi, Tamale and Sekondi–Takoradi) | Western Africa | Mixed-method (cross-sectional survey, in-depth interviews, field notes) | Single author: Ghana | No funding reported | - Trotro^ii^ - Taxi^vi^ |
| 4 | Kumar, 2011 ([Kumar, 2011](#_ENREF_38)) | Nigeria  (Lagos)  Cameroon  (Douala)  Uganda  (Kampala) | Western Africa  Central Africa  Eastern Africa | Mixed-method (cross-sectional survey, focus groups, semi-structured interviews, field notes) | Single author:  not reported | European Commission, Islamic Development Bank, African Development Bank, World Bank | - Minibus^ii^ - Shared taxi/van^ii^ - Commercial motorcycle^iv^ |
| 5 | Kola, 2012 ([Kola et al., 2012](#_ENREF_37)) | Kenya  (Kisumu) | Eastern Africa | Mixed-method (cross-sectional survey, land use maps, focus groups, semi-structured interviews, field notes) | First author: Kenya  Last author: Kenya | No funding reported | - Matatu^ii^ - Boda boda motorcycle taxi^iv^ - Boda boda bicycle taxi^viii^ - Tuk tuk^i^ |
| 6 | Diaz Olvera, 2013 ([Diaz Olvera et al., 2013](#_ENREF_17)) | Burkina Faso (Ouagadougou)  Cameroon  (Douala)  Guinea  (Conakry)  Mali  (Bamako)  Niger  (Niamey)  Senegal  (Dakar) | Central Africa  Central Africa  Western Africa  Western Africa  Western Africa  Western Africa | Mixed-method (cross-sectional survey, semi-structured interviews) | First author: France  Last author: France | No funding reported | - Motorbike taxi^iv^ - Shared taxi^vi^ - Minibus^ii^ - Midibus^ii^ |
| 7 | Vermeiren, 2015 ([Vermeiren et al., 2015](#_ENREF_76)) | Uganda  (Kampala) | Eastern Africa | Mixed-method  (cross-sectional survey, semi-structured interviews, field notes, participatory mapping) | First author: Belgium  Last author: Belgium | Belgium Fund for Scientific Research Flanders | - Matatu (minivan taxi)^ii^ - Boda boda (motorbike taxi)^iv^ |
| 8 | Mbara, 2016 ([Mbara, 2016](#_ENREF_45)) | South Africa (Johannesburg) | Southern Africa | Mixed-method (cross-sectional survey, unstructured interviews) | Single author: South Africa | No funding reported | - Tuk-tuk (auto-rickshaw)^i^ - Minibus taxi^ii^ |
| 9 | Andreasen, 2017 ([Andreasen & Møller-Jensen, 2017](#_ENREF_3)) | Tanzania  (Dar es Salaam) | Eastern Africa | Mixed-method (GIS analysis, focus groups, semi-structured interviews, key informant interviews, field notes) | First author: Denmark  Last author: Denmark | Rurban Africa funded by the European Union | - Daladala^ii^ - Bodaboda (motorcycle taxi)^iv^ - Bajaj (three-wheel scooter taxi)^i^ |
| 10 | Oviedo, 2017 ([Oviedo et al., 2017](#_ENREF_57)) | Nigeria  (Abuja, Kaduna and Ibadan) | Western Africa | Mixed-method (cross-sectional survey, semi-structured interviews, in-depth interviews) | First author: United Kingdom  Last author: United Kingdom | United Kingdom Department for International Development; ICF International; STO Associates, Nigeria | - Okada (motorcycle taxi)^iv^ - Danfo (minibus)^ii^ - Keke napep (rickshaw)^i^ - Shared taxi^vi^ |
| 11 | Porter, 2017 ([Porter et al., 2017](#_ENREF_60)) | South Africa  (Eastern Cape and Gauteng Province) | Southern Africa | Mixed-method (cross-sectional survey, in-depth interviews, focus groups, life histories, accompanied walks) | First author: United Kingdom  Last author: South Africa | United Kingdom Department for International Development; United Kingdom Economic and Social Research Council | - Minibus taxi^ii^ |
| 12 | Evans, 2018 ([Evans et al., 2018](#_ENREF_22)) | Uganda  (Kampala) | Eastern Africa | Mixed-method (cross-sectional survey, GIS analysis, semi-structured interviews, photo elicitation) | First author: United Kingdom  Last author: Germany | National Geographic | - Boda-boda motorcycle taxi^iv^ - Matatu^ii^ |
| 13 | Irlam, 2018 ([Irlam & Zuidgeest, 2018](#_ENREF_31)) | South Africa (Masiphumelele) | Southern Africa | Mixed-method (cross-sectional survey, focus groups) | Single author: South Africa | No funding reported | - Taxi^ii^ |
| 14 | Janusz, 2019 ([Janusz et al., 2019](#_ENREF_32)) | Uganda  (Kampala) | Eastern Africa | Mixed-method (GIS analysis, semi-structured interviews) | First author: Belgium  Last author: Belgium | No funding reported | - Matatu^ii^ - Boda-boda motorcycle taxi^iv^ - Boda-boda bicycle taxi^viii^ |
| Qualitative studies | | | | | | | |
| 1 | Kamuhanda, 2009 ([Kamuhanda & Schmidt, 2009](#_ENREF_33)) | Uganda  (Kampala) | Eastern Africa | Qualitative (structured interviews) | First author: Uganda  Last author: India | International Labor Organisation, United Kingdom Department for International Development | - Matatu^ii^ - Boda boda motorcycle taxi^iv^ - Car taxis (‘special hire’)^vi^ |
| 2 | Lucas, 2011 ([Lucas, 2011](#_ENREF_44)) | South Africa (Tshwane Metropolitan Region) | Southern Africa | Qualitative (focus groups) | Single author: United Kingdom | South African Department of Transport | - Minibus taxi or kombi^ii^ |
| 3 | Turner, 2012 ([Turner & Adzigbey, 2012](#_ENREF_71)) | Mozambique (Nampula)  Rwanda  (Kigali) | Eastern Africa  Eastern Africa | Qualitative (group (household) discussions) | First author: United Kingdom  Last author: United Kingdom | No funding reported | - Boda boda motorcycle taxi^iv^ - Boda boda bicycle taxi^viii^ |
| 4 | Raynor, 2014 ([Raynor, 2014](#_ENREF_62)) | Uganda  (Kampala) | Eastern Africa | Qualitative (focus groups, in-depth interviews) | Single author: Uganda | No funding reported | - Boda boda motorcycle taxi^iv^ - Matatu (metatu) omnibus^ii^ |
| 5 | Alando, 2016 ([Alando & Scheiner, 2016](#_ENREF_2)) | Kenya  (Kisumu) | Eastern Africa | Qualitative (focus groups, in-depth interviews) | First author: Germany  Last author: Germany | German Academic Exchange Program; Kenya National Council for Science, Technology and Innovation | - Boda boda motorcycle taxi^iv^ |
| 6 | Yankson, 2017 ([Yankson et al., 2017](#_ENREF_78)) | Ghana  (Sekondi–Takoradi) | Western Africa | Qualitative (focus groups, semi-structured interviews) | First author: Ghana  Last author: Ghana | Rurban Africa funded by the European Union | - Trotro minibus^ii^ |
| 7 | Lestevan, 2018 ([Lesteven & Boutueil, 2018](#_ENREF_42)) | Ethiopia  (Addis Ababa)  Kenya  (Nairobi)  South Africa  (Cape Town) | Eastern Africa  Eastern Africa  Southern Africa | Qualitative (semi-structured interviews, field notes) | First author: France  Last author: France | Sustainable Mobility Institute Renault-Paris Tech, as part of NexMob research project undertaken by City Mobility Transport Lab | - Matatu^ii^ - Minibus taxi^ii^ - Boda-boda motorbike taxi^iv^ - Saloon taxi^vi^ - Wuyeyet minibus taxi^ii^ - Midibus^ii^ |
| 8 | Poku-Boansi, 2018 ([Poku-Boansi & Cobbinah, 2018](#_ENREF_58)) | Ghana  (Kumasi) | Western Africa | Qualitative (semi-structured interviews) | First author: Ghana  Last author: Ghana | No funding reported | - Trotro (minibus taxi)^ii^ |
| ^i^ motorized three-wheelers; ^ii^ minibus/midibus taxis; ^iii^ adapted passenger vehicles built on a truck chassis; ^iv^ motorbike taxis; ^v^ non-motorized three-wheelers; ^vi^ shared sedan taxis; ^vii^ horse-drawn cart taxis; ^viii^ bicycle taxis  GIS – geographic information system | | | | | | | |
