# Supplementary material for: Active travel and paratransit use in African cities: Mixed-method systematic review and meta-ethnography
Source: J Transp Health. 2023 Jan;28:101558. doi: 10.1016/j.jth.2022.101558 (PMC9902334; doi:10.1016/j.jth.2022.101558)
Supplement: Multimedia component 3 [file mmc3.docx]

**Supplementary File 1: Eligible African countries with 2020 population figures in millions (rounded up or down to the nearest 100,000)**

| 1 | Algeria | 45,7 | 28 | Liberia | 5,1 |
| --- | --- | --- | --- | --- | --- |
| 2 | Angola | 36,9 | 29 | Libya | 6,9 |
| 3 | Benin | 12,1 | 30 | Madagascar | 27,7 |
| 4 | Botswana | 2,4 | 31 | Malawi | 19,1 |
| 5 | Burkina Faso | 20,9 | 32 | Mali | 20,3 |
| 6 | Burundi | 11,9 | 33 | Mauritania | 4,7 |
| 7 | Cabo Verde | 0,6 | 34 | Mauritius | 1,3 |
| 8 | Cameroon | 26,6 | 35 | Morocco | 36,9 |
| 9 | Central African Republic | 4,8 | 36 | Mozambique | 31,3 |
| 10 | Chad | 16,4 | 37 | Namibia | 2,5 |
| 11 | Comoros | 0,9 | 38 | Niger | 24,2 |
| 12 | Congo | 5,5 | 39 | Nigeria | 206,1 |
| 13 | Côte d’Ivoire | 26,4 | 40 | Rwanda | 13,0 |
| 14 | Democratic Republic of Congo | 89,6 | 41 | São Tomé and Príncipe | 0,2 |
| 15 | Djibouti | 1,0 | 42 | Senegal | 16,7 |
| 16 | Egypt | 102,3 | 43 | Seychelles | 0,01 |
| 17 | Equatorial Guinea | 1,4 | 44 | Sierra Leone | 8,0 |
| 18 | Eritrea | 3,6 | 45 | Somalia | 15,9 |
| 19 | Eswatini | 1,2 | 46 | South Africa | 59,3 |
| 20 | Ethiopia | 115,0 | 47 | South Sudan | 11,2 |
| 21 | Gabon | 2,2 | 48 | Sudan | 43,9 |
| 22 | Gambia | 2,4 | 49 | Tanzania | 59,7 |
| 23 | Ghana | 31,1 | 50 | Togo | 11,2 |
| 24 | Guinea-Bissau | 2,0 | 51 | Tunisia | 11,9 |
| 25 | Guinea | 13,1 | 52 | Uganda | 45,7 |
| 26 | Kenya | 53,8 | 53 | Zambia | 18,4 |
| 27 | Lesotho | 2,1 | 54 | Zimbabwe | 14,9 |

Source: <https://www.worldometers.info/geography/how-many-countries-in-africa/>
