# Supplementary material for: Active travel and paratransit use in African cities: Mixed-method systematic review and meta-ethnography
Source: J Transp Health. 2023 Jan;28:101558. doi: 10.1016/j.jth.2022.101558 (PMC9902334; doi:10.1016/j.jth.2022.101558)
Supplement: Supplementary Table 1 — Paratransit types reflected in the studies, with study regions shown. [file mmc4.docx]

| Studies  (arranged by year of publication and colour-coded by region) | No. of types mentioned | MOTORIZED TYPES | | | | | NONMOTORIZED TYPES | | |
| --- | --- | --- | --- | --- | --- | --- | --- | --- | --- |
|  |  | Minibus/ midibus taxis | Motor-bike taxis | Motorized  3-wheelers | Shared sedan  taxis | Adapted trucks | Bicycle taxis | Non-motor-  ized 3-wheelers | Horse-drawn cart taxis |
| Aworemi 2008 ([Aworemi et al., 2008](#_ENREF_5)) | 2 | X |  |  |  | X |  |  |  |
| Kamuhanda 2009 ([Kamuhanda & Schmidt, 2009](#_ENREF_33)) | 3 | X | X |  | X |  |  |  |  |
| Behrens 2010 ([Behrens & Schalekamp, 2010](#_ENREF_8)) | 1 | X |  |  |  |  |  |  |  |
| Diaz Olvera 2010 ([Diaz Olvera et al., 2010](#_ENREF_19)) | 2 | X | X |  |  |  |  |  |  |
| Elfiky 2010 ([Elfiky, 2010](#_ENREF_20)) | 2 | X |  | X |  |  |  |  |  |
| ITP 2010 ([Integrated Transport Planning Ltd., 2010](#_ENREF_30)) | 1 | X |  |  |  |  |  |  |  |
| Salon 2010 ([Salon & Gulyani, 2010](#_ENREF_66)) | 1 | X |  |  |  |  |  |  |  |
| Abane 2011 ([Abane, 2011](#_ENREF_1)) | 1 | X |  |  |  |  |  |  |  |
| Bwire 2011 ([Bwire, 2011](#_ENREF_11)) | 1 | X |  |  |  |  |  |  |  |
| Kumar 2011 ([Kumar, 2011](#_ENREF_38)) | 2 | X | X |  |  |  |  |  |  |
| Lucas 2011 ([Lucas, 2011](#_ENREF_44)) | 1 | X |  |  |  |  |  |  |  |
| Muhammed 2011 ([Muhammed, 2011](#_ENREF_50)) | 1 |  |  | X |  |  |  |  |  |
| Mutiso 2011 ([Mutiso & Behrens, 2011](#_ENREF_51)) | 4 | X | X | X |  |  | X |  |  |
| Venter 2011 ([Venter & Badenhorst, 2011](#_ENREF_75)) | 1 | X |  |  |  |  |  |  |  |
| Kola 2012 ([Kola et al., 2012](#_ENREF_37)) | 4 | X | X | X |  |  | X |  |  |
| Nwaogbe 2012 ([Nwaogbe et al., 2012](#_ENREF_53)) | 2 |  | X | X |  |  |  |  |  |
| Salon 2012 ([Salon & Aligula, 2012](#_ENREF_65)) | 1 | X |  |  |  |  |  |  |  |
| Turner 2012 ([Turner & Adzigbey, 2012](#_ENREF_71)) | 2 |  | X |  |  |  | X |  |  |
| Diaz Olvera 2013 ([Diaz Olvera et al., 2013](#_ENREF_17)) | 3 | X | X |  | X |  |  |  |  |
| Raynor 2014 ([Raynor, 2014](#_ENREF_62)) | 2 | X | X |  |  |  |  |  |  |
| Vermeiren 2015 ([Vermeiren et al., 2015](#_ENREF_76)) | 1 | X |  |  |  |  |  |  |  |
| Weinstock 2015 ([Weinstock et al., 2015](#_ENREF_77)) | 1 | X |  |  |  |  |  |  |  |
| Alando 2016 ([Alando & Scheiner, 2016](#_ENREF_2)) | 1 |  | X |  |  |  |  |  |  |
| Diaz Olvera 2016 ([Diaz Olvera et al., 2016](#_ENREF_18)) | 4 | X | X |  | X |  |  |  | X |
| Mbara 2016 ([Mbara, 2016](#_ENREF_45)) | 2 | X |  | X |  |  |  |  |  |
| Olawole 2016 ([Olawole & Olapoju, 2016](#_ENREF_55)) | 1 |  | X |  |  |  |  |  |  |
| Saddier 2016 ([Saddier et al., 2016](#_ENREF_64)) | 1 | X |  |  |  |  |  |  |  |
| Andreasen 2017 ([Andreasen & Møller-Jensen, 2017](#_ENREF_3)) | 3 | X | X | X |  |  |  |  |  |
| McKay 2017 ([McKay et al., 2017](#_ENREF_46)) | 1 | X |  |  |  |  |  |  |  |
| Oviedo 2017 ([Oviedo et al., 2017](#_ENREF_57)) | 4 | X | X | X | X |  |  |  |  |
| Porter 2017 ([Porter et al., 2017](#_ENREF_60)) | 1 | X |  |  |  |  |  |  |  |
| Sabry 2017 ([Sabry et al., 2017](#_ENREF_63)) | 2 |  |  | X |  |  |  | X |  |
| Yankson 2017 ([Yankson et al., 2017](#_ENREF_78)) | 1 | X |  |  |  |  |  |  |  |
| Chakwizira 2018 ([Chakwizira et al., 2018](#_ENREF_12)) | 1 | X |  |  |  |  |  |  |  |
| Evans 2018 ([Evans et al., 2018](#_ENREF_22)) | 2 | X | X |  |  |  |  |  |  |
| Irlam 2018 ([Irlam & Zuidgeest, 2018](#_ENREF_31)) | 1 | X |  |  |  |  |  |  |  |
| Lestevan 2018 ([Lesteven & Boutueil, 2018](#_ENREF_42)) | 3 | X | X |  | X |  |  |  |  |
| Poku-Boansi 2018 ([Poku-Boansi & Cobbinah, 2018](#_ENREF_58)) | 1 | X |  |  |  |  |  |  |  |
| Scorcia 2018 ([Scorcia & Munoz-Raskin, 2018](#_ENREF_67)) | 1 | X |  |  |  |  |  |  |  |
| Tembe 2018 ([Tembe et al., 2018](#_ENREF_68)) | 1 | X |  |  |  |  |  |  |  |
| Janusz 2019 ([Janusz et al., 2019](#_ENREF_32)) | 3 | X | X |  |  |  | X |  |  |
| **No. of studies** |  | n=35 | n=18 | n=9 | n=6 | n=4 | n=1 | n=1 | n=1 |
| **% of all studies** |  | 85% | 44% | 22% | 15% | 10% | 2% | 2% | 2% |
| Colour codes: Northern Africa, Western Africa, Eastern Africa, Southern Africa (multi-regional studies are uncoloured) | | | | | | | | | |
